# The *Megophthalmidia* (Diptera, Mycetophilidae) of North America including eight new species

**DOI:** 10.3897/zookeys.386.6913

**Published:** 2014-03-07

**Authors:** Peter H. Kerr

**Affiliations:** 1 California State Collection of Arthropods, California Department of Food and Agriculture, 3294 Meadowview Rd., Sacramento, CA, 95832–1448 USA

**Keywords:** Systematics, Leiinae, fungus gnats, new species, biodiversity hotspot, California State Parks, Annadel State Park, Calaveras Big Trees State Park, Humboldt Bay National Wildlife Refuge, Indian Grinding Rock State Historical Park, Point Reyes National Seashore, Prairie Creek State Park, University of California Sedgwick Reserve, University of California Donald and Sylvia McLaughlin Reserve, University of California Kenneth S. Norris Rancho Marino Reserve, University of California Whitaker Forest

## Abstract

*Megophthalmidia* Dziedzicki is a small leiine genus (Mycetophilidae) with seven species described from the Neotropics and ten species from the Palearctic region. Two species of *Megophthalmidia* have been reported for North America. Recent collecting of Mycetophilidae in California and Arizona, however, shows current North American diversity of *Megophthalmidia* is at least on par to other regions of the world. Eight new species of *Megophthalmidia* are described here, increasing the number of Nearctic *Megophthalmidia* species to nine. Included is a particularly atypical member of the genus, *M. saskia* **sp. n.**, which expands the genus concept of *Megophthalmidia*. Of the two species previously recorded for North America, only one actually belongs in the genus. *Megophthalmidia occidentalis* Johannsen, is fully described and illustrated. The other named species, *M. marceda* (Sherman) is illustrated and transferred to the genus *Ectrepesthoneura* Enderlein. A lectotype is designated for this species. A key to the species of *Megophthalmidia* of North America is provided. The biology of these flies is not yet known. Three of the new *Megophthalmidia* species – *M. lenimenta*, *M. misericordia*, and *M. radiata* – are only known to occur within small protected areas within the California State Park and UC Natural Reserve systems.

## Introduction

*Megophthalmidia* Dziedzicki is a small leiine genus (Mycetophilidae) with seven species described from the Neotropics and ten species from the Palearctic region (Bechev 1999; Chandler et al. 2006). Two species of *Megophthalmidia* Dziedzicki have been reported to occur in North America, however both remain very poorly characterized. The description of *Megophthalmidia occidentalis* Johannsen, 1909, originally from Washington State, is a footnote – literally – consisting of a little more than one line of text (Johannsen 1909). For many years, this was the only *Megophthalmidia* species known to occur in North America and as a consequence, *Megophthalmidia* specimens captured in USA and Canada were reflexively identified as *Megophthalmidia occidentalis* (similar to the case of *Acomoptera plexipus* (Garrett) (Kerr 2011)). The other Neactic species ascribed to the genus, *Megophthalmidia marceda* (Sherman, 1921), was considered a member of *Tetragoneura* Winnertz until Coher transferred it more than 75 years later (Coher 1999). Unfortunately, neither species has been treated in depth and the only available descriptions and illustrations are wholly inadequate for their proper identification. In light of this, and the discovery of additional species in our region, the diagnostic features of North American *Megophthalmidia* are figured and compared. A key to the species is also provided.

In the Nearctic, commonly found members of *Megophthalmidia* and *Aphrastomyia* Coher & Lane are regularly confused for one another on account of their very similar body form and wing venation. The chapter on Mycetophilidae in the Nearctic Manual (Vockeroth 1981) doesn’t particularly help clarify the genera in this regard, since at the beginning of the key, *Aphrastomyia* is separated by a character state that is also present in species of *Megophthalmidia* (viz., fine tibial setae arranged in regular longitudinal rows). Despite their similarities, *Megophthalmidia* and *Aphrastomyia* are currently not considered sister taxa. Oliveira (2013) recently treated the Leiinae and in her study, *Aphrastomyia* and the monotypic Afrotropical genus, *Mohelia* Matile, were recovered together. *Megophthalmidia*, in turn, was recovered as sister to these genera (*Megophthalmidia* + (*Aphrastomyia* + *Mohelia*)) (Oliveira 2013). These results are consistent with the intuitive hypotheses of relationship for this group presented by Matile (1978) and Jaschhof and Kallweit (2004).

Included in the new material from California is a new species that retains affinities to both *Megophthalmidia* and *Mohelia*. The recognition of this new species has implications for current generic concepts and may possibly alter our understanding of the history and relationships between lineages related to and including *Megophthalmidia*.

This paper presents new species of *Megophthalmidia* found in the Nearctic Region and discusses the limits of the genus, in its current form, in North America. A phylogenetic treatment of this genus and its nearest relatives worldwide is outside the scope of this paper, but the subject of ongoing research as additional material is accumulated.

## Materials and methods

Terminology for wing venation generally follows McAlpine (1981), however interpretation of radial veins is consistent with Söli (1997). Terminology for thoracic and genital morphology largely follows Wood (1991), McAlpine (1981), Vockeroth (1981), and Kerr (2011); the terms “genitalia” and “terminalia” are used interchangeably. Genitalia were macerated in 10% KOH at approx. 95 °C for 15–20 minutes to remove soft tissue, then rinsed in distilled water and dilute glacial acetic acid, and dissected in water. All genitalia preparations were placed in a small genitalia vial containing glycerol, and pinned beneath the specimen. Figures were made using Adobe Illustrator and Adobe Photoshop from Adobe Creative Suite 5 software, with digital images taken using a Nikon DS-Fi1 scope-mounted digital camera. Habitus images were taken with the same digital camera (or the Nikon DS-Fi2), using an LED dome lighting system (Kerr et al. 2008). Material examined includes holdings deposited in the California Academy of Sciences, San Francisco, CA (CAS); California State Collection of Arthropods, Sacramento, CA (CSCA); Canadian National Collection, Ottawa, Ontario (CNC); Cornell University Insect Collection, Ithaca, NY (CUIC); Los Angeles County Museum of Natural History, Los Angeles, CA (LACM); Santa Barbara Museum of Natural History, Santa Barbara, CA (SBNM); and University of Arizona Insect Collection, Tuscon, AZ (UAIC). Specific collection holding and deposition information is provided in the species accounts, in square brackets after the transcribed specimen label data.

All measurements are made in millimeters. Ranges are given for body length, wing length, and the mean for each of these values is provided. Measurements of holotypes are given in square brackets. The number of individuals measured is noted in parentheses. All measurements are of critical-point dried specimens. Most females were not found to be diagnostic at the species level, however based on matching color patterns and simultaneous collecting events, females were linked to conspecific males. Representative habitus images for these females are provided.

### Key to the *Megophthalmidia* of North America (males)

**Table:** 

1	Thorax yellow, light-colored dorsum contrasting with dark brown head (Fig. 51), aedeagus as Figs 59–61	*Megophthalmidia occidentalis* Johannsen
–	Thorax brown or black, dorsum concolorous with head, aedeagus variable	2
2	Frons bearing setae; CuP present as lightly sclerotized fold (Fig. 83); epandrium with stout setae at posterior margin (Figs 84, 86), epandrial lobes absent	*Megophthalmidia saskia* sp. n.
–	Frons without setae; wing vein CuP absent (as Fig. 52); apical epandrial lobes present (e.g., Figs 2, 3, 12, 13)	3
3	Apical epandrial lobes elongate, at least 5 × longer than width at base (Figs 3, 23, 33)	4
–	Apical epandrial lobes stouter, 4 × longer (or less) than width at base (Figs 13, 43, 64, 74)	6
4	Apical epandrial lobes slightly swollen at apex (wider than midpoint width), often curving outward (Fig. 33); posterior margin of epandrium evenly tapered inward, without strong medial notch (Fig. 34)	*Megophthalmidia mckibbeni* sp. n.
–	Apical epandrial lobes at apex as wide as midpoint width, oriented in downward in line with rest of lobe (Figs 3, 23); posterior margin of epandrium with strong medial notch (Figs 4, 24)	5
5	Aedeagal process with upward recurved tine (Figs 8, 10), dorsomedial surface of epandrium less than half epandrial length (Fig. 4)	*Megophthalmidia browni* sp. n.
–	Aedeagal process without upward recurved tine (Figs 28, 30), dorsomedial surface of epandrium at least half of epandrial length (Fig. 24)	*Megophthalmidia lenimenta* sp. n.
6	Surface of apical epandrial lobe setose (Fig. 43)	*Megophthalmidia misericordia* sp. n.
–	Surface of apical epandrial lobe lacking setae (Figs 13, 64, 74)	6
7	Apical epandrial lobes thickened at base (Figs 73, 74); short tine of adeagal fork much thinner than long tine, pointed upward (Fig. 79)	*Megophthalmidia radiata* sp. n.
–	Apical epandrial lobes not thickened at base or thickened only slightly (Figs 12, 13, 63, 64); short tine of aedeagal fork thicker than long tine, pointed outward (Figs 19, 70)	8
8	Apical epandrial lobes relatively short, approx. 2× longer than wide, turned inward so appearing as wide in posterior view as in lateral view (Figs 63, 64); diameter of short tine of adeagal fork less than 2× diameter of long tine at base (Fig. 70)	*Megophthalmidia perignea* sp. n.
–	Apical epandrial lobes not as short, approx. 2.5× longer than wide, appearing wider in posterior view than in lateral view (Figs 12, 13); diameter of short tine of adeagal fork approx. 3× diameter of long tine at base (Fig. 19)	*Megophthalmidia ignea* sp. n.

## Data resources

The data underpinning the analyses reported in this paper are deposited in GBIF, the Global Biodiversity Information Facility, http://ipt.pensoft.net/ipt/resource.do?r=megophthalmidia_of_north_america.

## Taxonomy

### Megophthalmidia browni sp. n.

http://zoobank.org/D35852D1-4B12-4360-8565-54AC77009DE3

http://species-id.net/wiki/Megophthalmidia_browni

**Figure 1.**
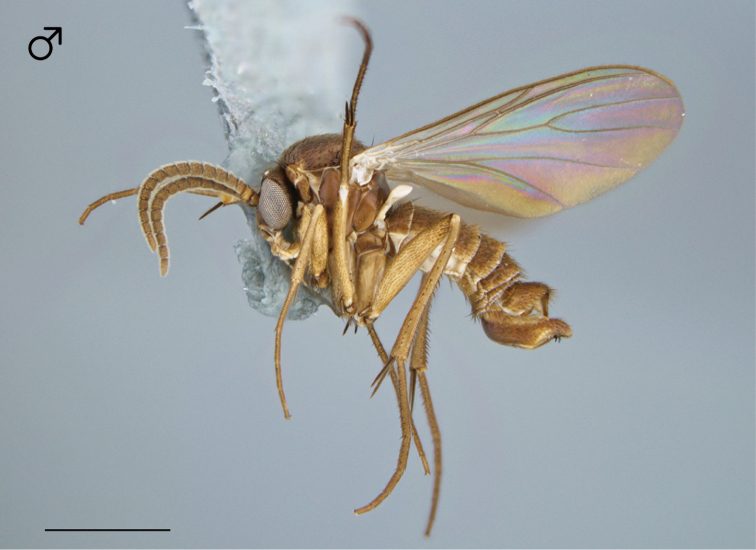
*Megophthalmidia browni* sp. n., habitus [holotype male, # 12K450; female unknown]. Scale bar = 1 mm.

Figs 1 [Fig F2] [Fig F3] –10

#### Type material.

Holotype: ♂, “USA: CA: Santa Barbara Co., UC Sedgwick Reserve, Malaise, 34.6853°N,-120.0461°W, 1–5.ii.2005 M. Caterino CSCA12L353” / “HOLOTYPE 12K450, *Megophthalmidia browni* ♂, Kerr, 2014” [red label]. Deposited in CSCA, mounted on gray point, complete specimen in good condition (Fig. 1).

Paratypes (all bearing a blue paratype label): ♂, “USA: CA: Los Angeles Co., Brentwood 34.07°N, 118.49°W, 2–20.iii.2008 M. Schulman, MT, backyard garden CSCA08L642” [CSCA; locality Fig. 105, specimen # 12J952 (dissected, Figs 2-10)]; ♂, “USA: CA: Los Angeles Co., Brentwood 34.07°N, 118.49°W, 20.iii–9.iv.2008 M. Schulman, MT, backyard garden CSCA08L640” [CSCA; specimen # 09E060].

Additional material examined: 3 ♂♂, “MEX. Baja Calif. Norte, Arr. Santo Domingo, 5.7mi E. Hamilton Ranch / dam site, 18–IV–1963, H.B. Leech, P.H. Arnaud, Jr.” [CAS; one specimen dissected, specimen # 13M591].

#### Diagnosis.

**Figures 2–4. F2:**
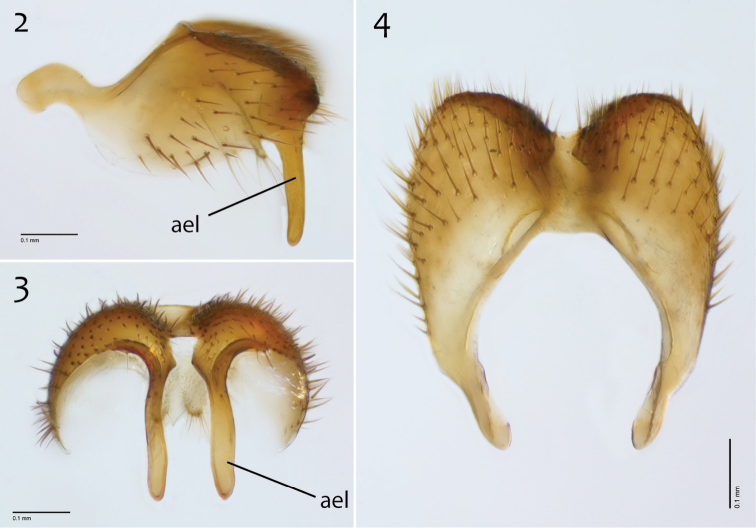
*Megophthalmidia browni* sp. n., male epandrium [paratype, # 12J952] **2** Lateral view **3** Posterior view **4** Dorsal view. Scale bar = 0.1 mm. Abbreviations: **ael** apical epandrial lobe.

*Megophthalmidia browni* sp. n. is most similar to *Megophthalmidia lenimenta* sp. n. having epandria that have a medial furrow and central notch, with slender apical processes. In *Megophthalmidia browni*, the apical epandrial processes are longer (Fig. 2) than in *Megophthalmidia lenimenta* (Fig. 22), but differences between the species are more obvious in the aedeagal morphology. *Megophthalmidia browni* bears a recurved aedeagal fork (Fig. 8) whereas in *Megophthalmidia lenimenta*, this structure is lacking (Fig. 28). *Megophthalmidia browni* may be distinguished from *Megophthalmidia ignea* and*Megophthalmidia perignea* by the shape of the apical epandrial processes (narrow elongate, as opposed to shortened) and from *Megophthalmidia mckibbeni* by the distinctive invagination of the apical epandrial processes at their base and the presence of a dorsally-reflexed bifurcation of the aedeagal fork (absent in *Megophthalmidia mckibbeni*). The aedeagal complex of *Megophthalmidia browni* displays bifurcating tines of approximately the same width, in which the shorter fork is directed anteriorly (Fig. 8).

#### Description.

Male. Body length: 2.7–2.8, 2.1 [2.7] mm (n=2). Wing length: 2.6–2.8, 2.7 [2.8] mm (n=3).

*Coloration* (Fig. 1). Head dark brown; antennal scape dark brown, pedicel brown or bearing some cream-color or pale yellow, and flagellomeres brown; face dark brown, clypeus and labrum brown to dark brown; palps and labellum cream-colored to pale yellow (palpomeres 1 and 2 usually slightly darker than others, palpomere 2 with light patch where sensilla present). Thorax brown to dark brown throughout, except at the anterolateral margin of the dorsum and dorsal pronotal area, where it is cream-colored or pale yellow; scutum setae golden brown to dark brown. Coxae clearly lighter in color than thorax, cream-colored to pale yellow; femora cream-colored to light brown throughout (except sometimes slightly brown at dorsal apex); tibiae and tarsi cream-colored to pale yellow, with densely-arranged dark brown setae; hind tibial comb yellowish, preceded by 0–3 (usually 3) dark brown setae. Wing hyaline without markings, wing veins brown; haltere stem and knob white to cream-colored. Abdominal segments light brown to brown, darker laterally. Terminalia light brown to brown.

*Head*. Ocelli slightly raised, median ocellus in line with anterior margin of lateral ocelli, median ocellus approx. 0.5× size of lateral ocelli; lateral ocellus located approx. 2× diameter of ocellus from eye margin, separated from median ocellus by approx. 2.3–2.8× its own diameter. Eyes with microsetae, which are approximately as long as width of facet. Frons microtrichose, without setae, flattened. Antennal length 1.4–1.6, 1.5 [1.6] mm (n=3). Face clearly longer than wide, setose; clypeus and labrum microtrichose, without setae. Palpus with four palpomeres; palpomere 1 oblong, without setae; other palpomeres with brown setae; palpomere 2 bearing small pocket of sensilla; palpomere 1 length longer than or subequal in length to palpomere 2; palpomere 3 length shorter than combined length of palpomeres 1 and 2; palpomere 4 subequal in length to combined length of palpomeres 2 and 3.

*Thorax*. Dorsum with evenly-distributed, short, appressed setae, bearing longer setae only along lateral and posterior margins. Antepronotum, proepisternum, and laterotergite bearing setae; remaining lateral thoracic sclerites bare. Costal wing vein extends beyond R_5_, approx. two-thirds distance between R_5_ and M_1_; R_1_ approximately the same length as r-m or slightly longer; cubital fork proximad of r-m base (as in *Megophthalmidia occidentalis*, Fig. 52); R_1_, M_1_, M_2_, CuA_1_, and CuA_2_ with setae on upper surface (lacking setae on M_1_ + M_2_). Wing veins A1 and CuP absent.

**Figures 5–7. F3:**
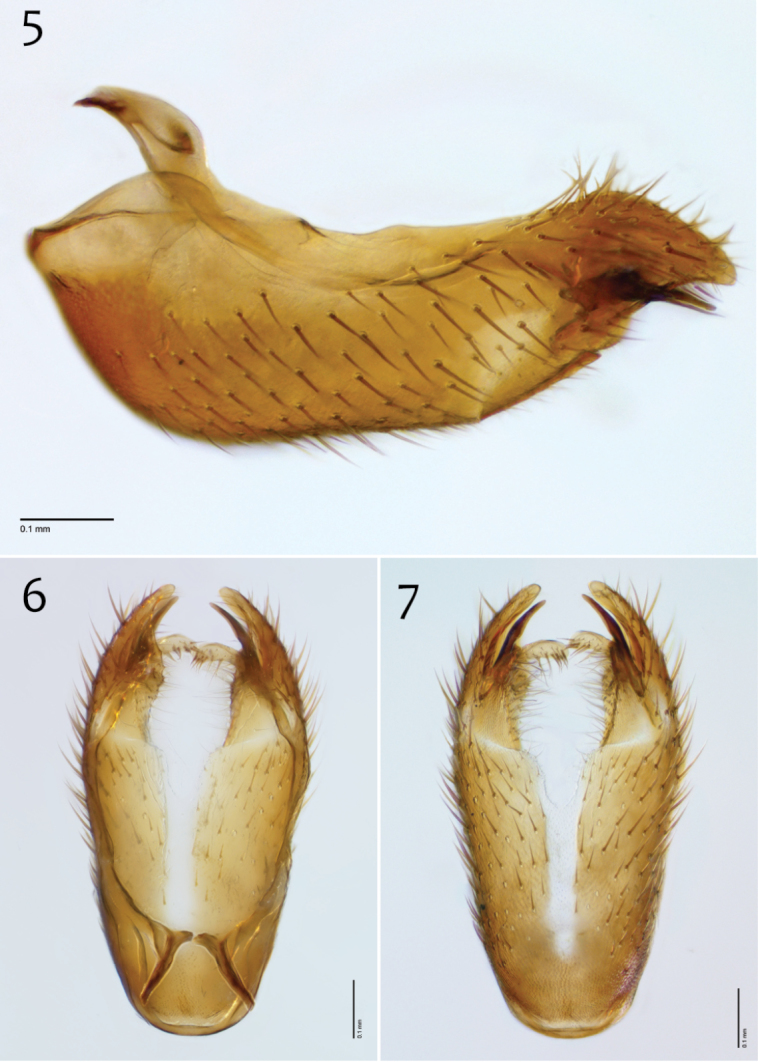
*Megophthalmidia browni* sp. n., male hypandrium [paratype, # 12J952] **5** Lateral view **6** Dorsal view **7** Ventral view. Scale bar = 0.1 mm.

**Figures 8–10.**
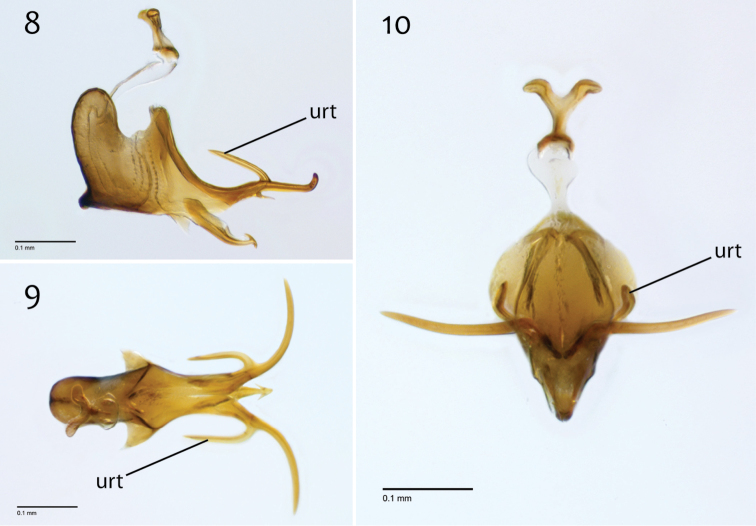
*Megophthalmidia browni* sp. n., male aedeagus [paratype, # 12J952] **8** Lateral view **9** Dorsal view **10** Posterior view. Scale bar = 0.1 mm. Abbreviations: **urt** upward recurved tine.

*Male genitalia* (Figs 2–10). Epandrium dorsal surface with clear medial depression, where setae are lacking; posterior margin narrowly emarginate at center (Fig. 4). Posterior processes of epandrium elongate, approx. 5–6× longer than wide, separated at base by approx. 1× width of process, length of setae at base of epandrial processes 2–3× width of process, bare along most of length (Figs 2, 3). Gonocoxites as in Figs 5–7. Aedeagal fork bifurcated into elongated tines of similar width; shorter tine recurved to point anteriorly, longer tine curved outward (Figs 8–10).

Female unknown.

#### Etymology.

The species epithet “browni” is a noun in the genitive case, named in honor of Brian V. Brown, friend, colleague, mentor, and Curator, Natural History Museum of Los Angeles County.

### Megophthalmidia ignea sp. n.

http://zoobank.org/0A125C12-EFB9-47BF-8F71-A70C946E365C

http://species-id.net/wiki/Megophthalmidia_ignea

**Figure 11.**
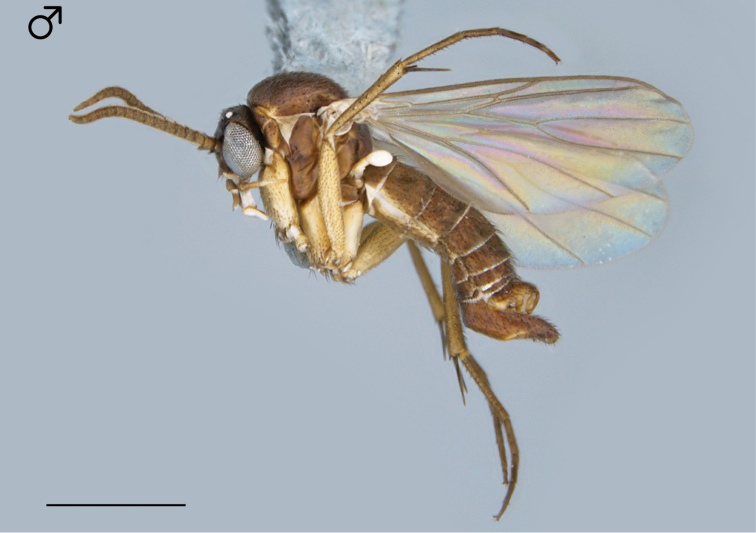
*Megophthalmidia ignea* sp. n., habitus [paratype male, # 12J388; female unknown]. Scale bar = 1 mm.

Figs 11 [Fig F6] [Fig F7] –20

#### Type material.

Holotype: ♂, “USA: CA: San Bernardino Co., 4 km SE Wrightwood, Lone Pine Canyon, 34°19.03'N, 117°34.93'W, elev. 5388’, 21–28.v.2005, S.L. Winterton & A.R. Cline, Malaise CDFA2005-008” / “HOLOTYPE 12J949, *Megophthalmidia ignea* ♂, Kerr, 2014” [red label]. Deposited in CSCA, mounted on gray point, missing hind left leg and mid right leg, otherwise in good condition; specimen dissected, terminalia preserved in glycerol in glass genitalia vial pinned beneath specimen. See Fig. 106 for image of type locality.

**Figures 12–14. F6:**
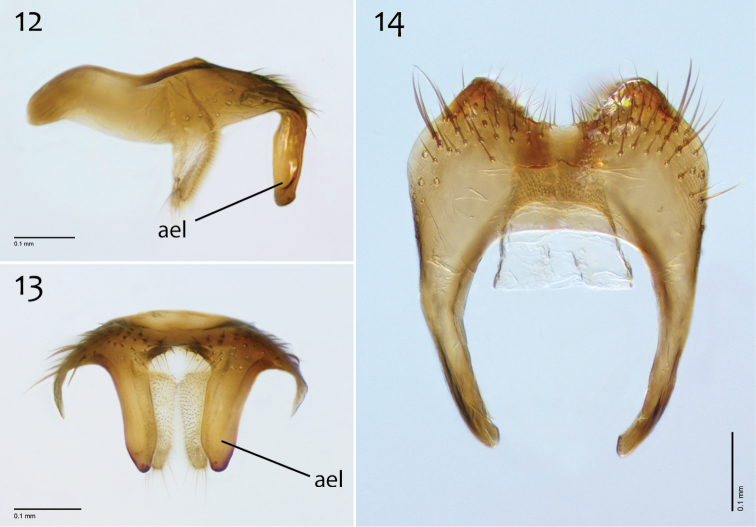
*Megophthalmidia ignea* sp. n., male epandrium [paratype, # 09C893] **12** Lateral view **13** Posterior view **14** Dorsal view. Scale bar = 0.1 mm. Abbreviations: **ael** apical epandrial lobe.

**Figures 15–17. F7:**
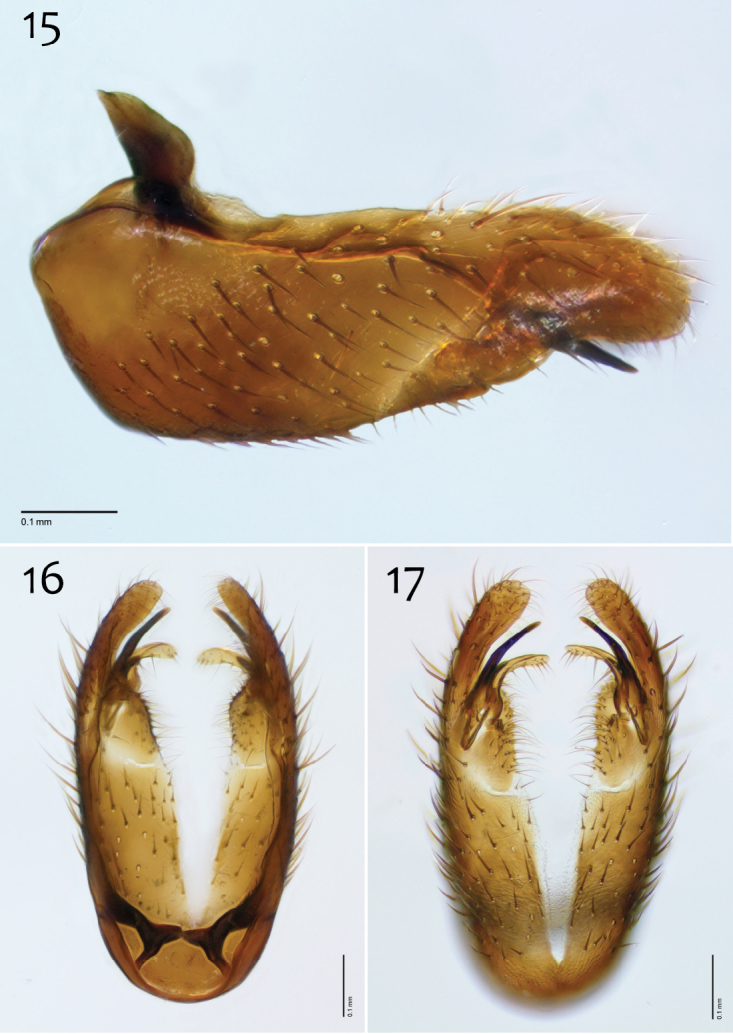
*Megophthalmidia ignea* sp. n., male hypandrium [paratype, # 09C893] **15** Lateral view **16** Dorsal view **17** Ventral view. Scale bar = 0.1 mm.

**Figures 18–20.**
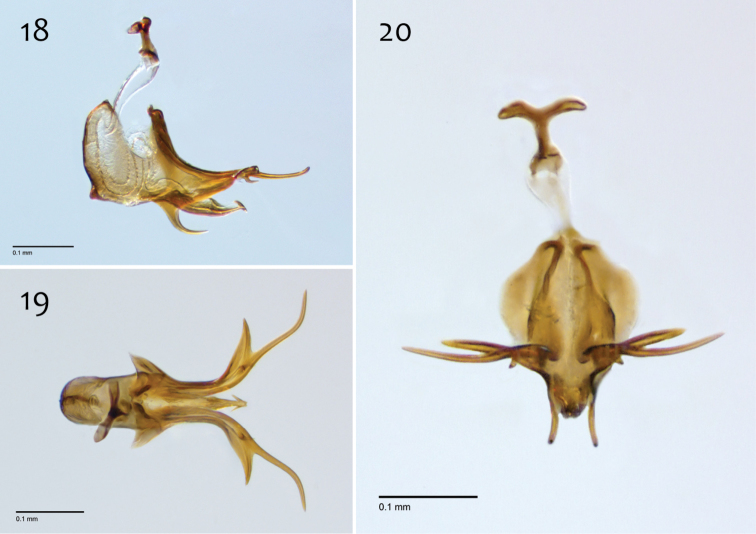
*Megophthalmidia ignea* sp. n., male aedeagus [paratype, # 09C893] 1**8** Lateral view **19** Dorsal view **20** Posterior view. Scale bar = 0.1 mm.

Paratypes (all bearing a blue paratype label): ♂, “USA: CA: Los Angeles Co., 13kmNW Wrightwood, Largo Vista Rd., 34°25.32'N, 117°46.06'W; elev. 6516’, 21–28.v.2005, S.L. Winterton & A.R. Cline, Malaise in dry wash, CDFA2005-001” [CSCA; specimen # 12J388 (Fig. 11)]; 3 ♂♂, “USA: CA: San Bernardino Co., SBNF: W. of Barton Flats, MT, 34.1677°N,-116.9146°W, 23–31.v.2004 M. Caterino CSCA12L335” [CSCA; specimen numbers 12K356, 13M304, and 13M319 (dissected)]; ♂, “USA: CA: San Bernardino Co. 4 km SE Wrightwood. Lone Pine Canyon, MT 34.317167° -117.582167°, elev. 5388’ 23–25.v.2009 S.L. Winterton CSCA09L313” [CSCA; specimen # 09C893 (Figs 12–20)].

#### Diagnosis.

*Megophthalmidia ignea* sp. n. may be confused with Nearctic congeners that also have a brown thorax contrasting against cream-colored tibia. Among these, it is similar to *Megophthalmidia browni* sp. n. and *Megophthalmidia mckibbeni* sp. n. but may be distinguished from these species by the shape and setation of the apical epandrial processes (Figs 12, 13); shortened and bare, as opposed to elongate in *Megophthalmidia browni* and setose in *Megophthalmidia mckibbeni*). Among congeners, however, it most resembles *Megophthalmidia perignea*, even in the general shape of the aedeagal complex. Most characteristically, the short aedeagal tine of *Megophthalmidia ignea* is very thick at its base, distinguishing itself from its sister taxon (Fig. 19). Also, in *Megophthalmidia ignea*, the apical epandrial processes are longer and more slender (Figs 12, 13) than in *Megophthalmidia perignea* (Figs 63, 64).

#### Description.

Male. Body length: 2.6–2.9, 2.8 [n/a] mm (n=4). Wing length: 2.5–2.9, 2.7 [2.9] mm (n=5).

*Coloration* (Fig. 11). Head dark brown; antennal scape dark brown, pedicel and flagellomeres brown; face dark brown, clypeus and labrum brown to dark brown; palps and labellum cream-colored to pale yellow (palpomeres 1–3 usually slightly darker than others, palpomere 2 with light patch where sensilla present). Thorax brown to dark brown throughout, except at the anterolateral margin of the dorsum and dorsal apronotal area, where it may be narrowly cream-colored or pale yellow; scutum setae golden brown to dark brown. Coxae clearly lighter in color than thorax, cream-colored to pale yellow; femora cream-colored to light brown throughout (sometimes slightly brown at dorsal apex), dark brown at apical margin; tibiae and tarsi cream-colored to pale yellow, with densely-arranged dark brown setae; hind tibial comb yellowish, preceded by 0–3 (usually 3) dark brown setae. Wing hyaline without markings, wing veins brown; haltere stem and knob white to cream-colored. Abdominal segments concolorous brown. Terminalia light brown to brown.

*Head*. Ocelli slightly raised, median ocellus in line with anterior margin of lateral ocelli, median ocellus approx. 0.3×–0.5× size of lateral ocelli; lateral ocellus located approx. 2× diameter of ocellus from eye margin, separated from median ocellus by approx. 2.2× its own diameter. Eyes with microsetae, which are approximately as long as width of facet. Frons microtrichose, without setae, flattened. Antennal length 0.9–1.3, 1.1 [1.3] mm (n=5). Face clearly longer than wide, setose; clypeus and labrum microtrichose, without setae. Palpus with four palpomeres; palpomere 1 oblong, without setae; other palpomeres with brown setae; palpomere 2 bearing small pocket of sensilla; palpomere 1 length longer than or subequal in length to palpomere 2; palpomere 3 approx. same length as combined length of palpomeres 1 and 2; palpomere 4 subequal or slightly shorter in length to combined length of palpomeres 2 and 3.

*Thorax*. Dorsum with evenly-distributed, short, appressed setae, bearing longer setae only along lateral and posterior margins. Antepronotum, proepisternum, and laterotergite bearing setae; remaining lateral thoracic sclerites bare. Costal wing vein extends beyond R_5_, approx. three-fifths distance between R_5_ and M_1_; R_1_ approximately the same length as r-m or slightly longer; cubital fork below or proximad of r-m base (as in *Megophthalmidia occidentalis*, Fig. 52); R_1_, M_1_, M_2_, CuA_1_, and CuA_2_ with setae on upper surface (lacking setae on M_1_ + M_2_). Wing veins A1 and CuP absent.

*Male genitalia* (Figs 12–20). In some specimens, terminalia distinctly procumbent. Posterior margin of epandrium broadly emarginate at center (Fig. 14). Posterior processes of epandrium relatively short, approx. 2.5× longer than wide, separated at base by approx. 1.75× width of process; bare (Figs 12, 13). Gonocoxites as in Figs 15–17. Aedeagal fork bifurcated into tines; shorter tine noticeably thickened (2–3× wider than paired tine at base), both tines pointed outward apically (Fig. 19).

Female unknown.

#### Etymology.

The species epithet “ignea” is an adjective, derived for the Latin word for “fiery” in reference to the typical chaparral habitat of this species, whose ecology is shaped by fire.

### Megophthalmidia lenimenta sp. n.

http://zoobank.org/65C644B7-D627-41F2-96F8-464E57B062E5

http://species-id.net/wiki/Megophthalmidia_lenimenta

**Figure 21.**
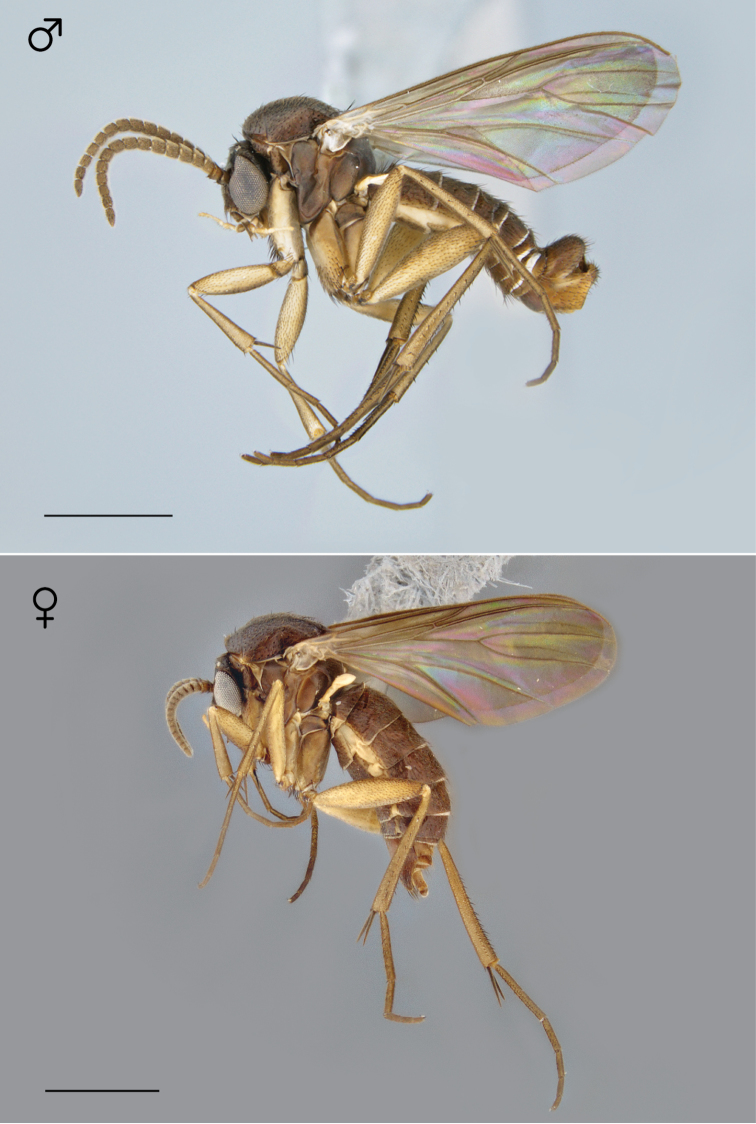
*Megophthalmidia lenimenta* sp. n., habitus [holotype male above, # 13M302; female below, # 13M339]. Scale bar = 1 mm.

Figs 21 [Fig F10] [Fig F11] –30

#### Type material.

Holotype: “USA: CA: Yolo Co., McLaughlin NR, Clover Valley, Oak grassland, MT#1, 38.8400°N, 122.3451°W, 500masl, 24.iii–29.iv.2010 P. H. Kerr & C. E. Koehler, CSCA10L050” / “HOLOTYPE 13M302, *Megophthalmidia lenimenta* ♂, Kerr, 2014” [red label]. Deposited in CSCA, mounted on gray point, complete specimen (Fig. 21). See Fig. 108 for image of type locality.

Paratypes (all bearing a blue paratype label): 3 ♂♂, 2♀♀, same locality as holotype [Locality Fig. 108; SBNM # 13M283 (♂); CSCA 13M317 (dissected ♂; Figs 22–30), 13M337 (♂),13M338 (♀), 13M339 (♀; Fig. 21)]; 3 ♂♂, ♀ “USA: CA: Yolo Co., McLaughlin NR, Clover Valley, Oak grassland, MT#1, 38.8400°N, 122.3451°W, 500masl, 29.iv–9.vi.2010 P. H. Kerr & C.E. Koehler, 10L187” [LACM # 11H156 (♂); CSCA numbers 13M303 (♂, dissected), 13M340 (♂), and 13M342 (♀)].

Additional material examined: 2 ♂♂, “USA: CALIFORNIA, Stanislaus County, Del Puerto Canyon, Frank Raines Park / ca 1120’, 3–IV–70, Paul H. Arnaud, Jr., Collector” [CAS; one specimen dissected, #13M588].

#### Diagnosis.

*Megophthalmidia lenimenta* sp. n. may be confused with several Nearctic congeners that also have a brown thorax. Among these, it is probably most similar to *Megophthalmidia browni* sp. n. on account of both species having epandria with a medial notch along the posterior margin, a medial depression, and elongated posterior processes. In *Megophthalmidia lenimenta*, one aedeagal tine is very reduced/undeveloped, only slightly longer than wide (Fig. 29; whereas in *Megophthalmidia browni* both tines are many times longer than wide (Fig. 9)). Among other Nearctic congeners with elongate posterior epandrial processes, including *Megophthalmidia browni*, *Megophthalmidia lenimenta* is also distinguished by having length of dorsomedial epandrial surface at least half the length of epandrium (Fig. 24).

#### Description.

Male. Body length: 2.4–3.1, 2.8 [n/a] mm (n=5). Wing length: 2.6–2.9, 2.8 [2.9] mm (n=7).

*Coloration* (Fig. 21). Male. Head dark brown; antennal scape brown or dark brown, pedicel and flagellomeres brown; face dark brown, clypeus and labrum brown to dark brown; palps and labellum cream-colored to pale yellow (palpomeres 1–3 usually slightly darker than others, palpomere 2 with light patch where sensilla present). Thorax brown to dark brown throughout; scutum setae brown. Coxae lighter in color than thorax, cream-colored to light brown, sometimes with area at base somewhat darker in color, fore coxa slightly lighter in color than mid- and hind coxa; femora cream colored to light brown, becoming gradually darker dorsoapically on mid- and hind femora; tibiae light brown, tarsi light brown to brown; hind tibial comb yellowish, preceded by 0–3 (usually 3) dark brown setae. Wing hyaline without markings, wing veins brown; haltere stem and knob white to cream-colored. Abdominal segments concolorous brown to dark brown. Terminalia brown.

*Head*. Ocelli slightly raised, median ocellus in line with anterior margin of lateral ocelli, median ocellus approx. 0.3–0.5× size of lateral ocelli; lateral ocellus located approx. 2× diameter of ocellus from eye margin, separated from median ocellus by approx. 1.9–2× its own diameter. Eyes with microsetae, which are approximately as long as width of facet. Frons microtrichose, without setae, flattened. Antennal length 1.3–1.7, 1.6 [1.5] mm (n=7). Face clearly longer than wide, setose; clypeus and labrum microtrichose, without setae. Palpus with four palpomeres; palpomere 1 oblong-triangular, without setae; other palpomeres with brown setae; palpomere 2 bearing small pocket of sensilla; palpomere 1 length longer than or subequal in length to palpomere 2; palpomere 3 length subequal to or slightly shorter than combined length of palpomeres 1 and 2; palpomere 4 length 0.7–1× combined lengths of palpomeres 1–3.

*Thorax*. Antepronotum, proepisternum, and laterotergite bearing setae; remaining lateral thoracic sclerites bare. Dorsum with evenly-distributed, short, appressed setae, bearing longer setae only along lateral and posterior margins. Costal wing vein extends beyond R_5_, approx. two-thirds distance between R_5_ and M_1_; R_1_ longer than r-m; cubital fork proximad of r-m base (as in *Megophthalmidia occidentalis*, Fig. 52); R_1_, M_1_, M_2_, CuA_1_, and CuA_2_ with setae on upper surface (lacking setae on M_1_ + M_2_). Wing veins A1 and CuP absent.

**Figures 22–24. F10:**
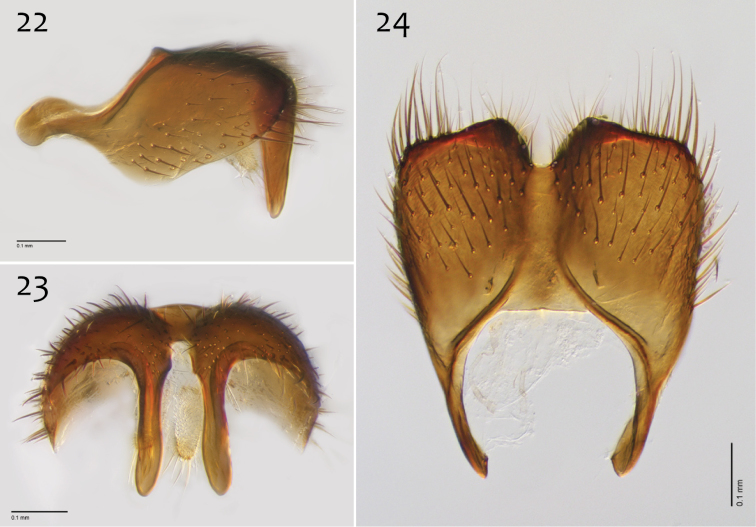
*Megophthalmidia lenimenta* sp. n., male epandrium [paratype, # 13M317] **22** Lateral view **23** Posterior view **24** Dorsal view. Scale bar = 0.1 mm.

**Figures 25–27. F11:**
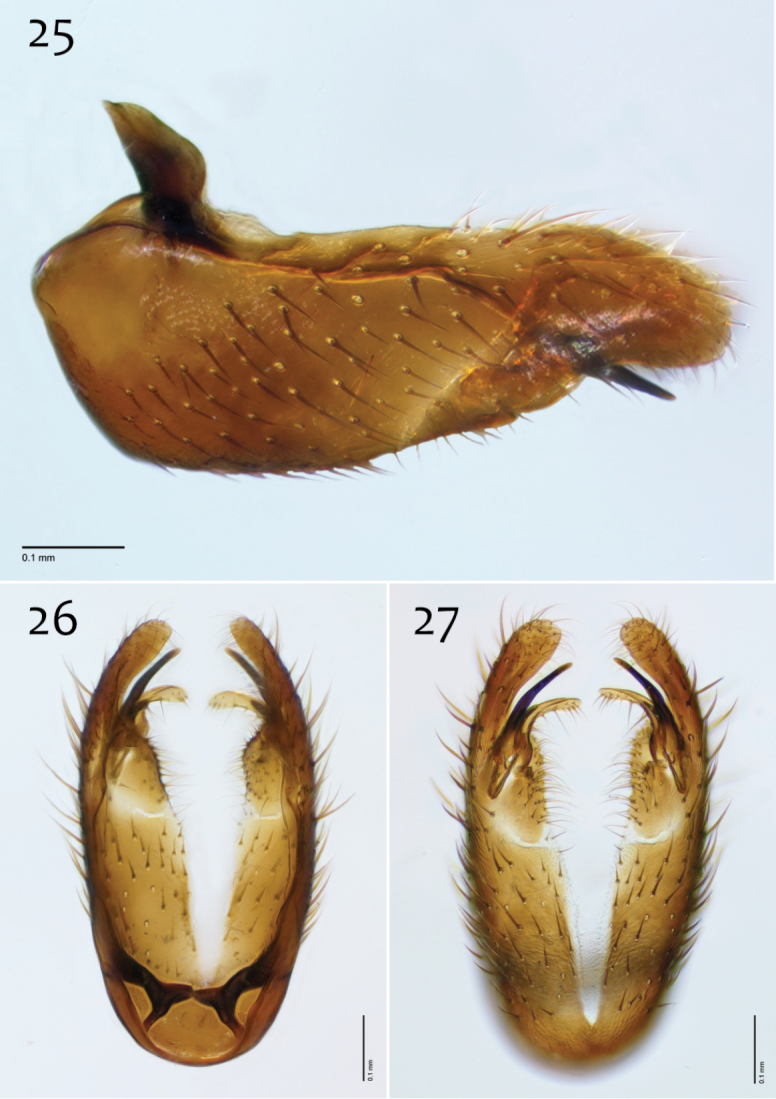
*Megophthalmidia lenimenta* sp. n., male hypandrium [paratype, # 13M317] **25** Lateral view **26** Dorsal view **27** Ventral view. Scale bar = 0.1 mm.

**Figures 28–30.**
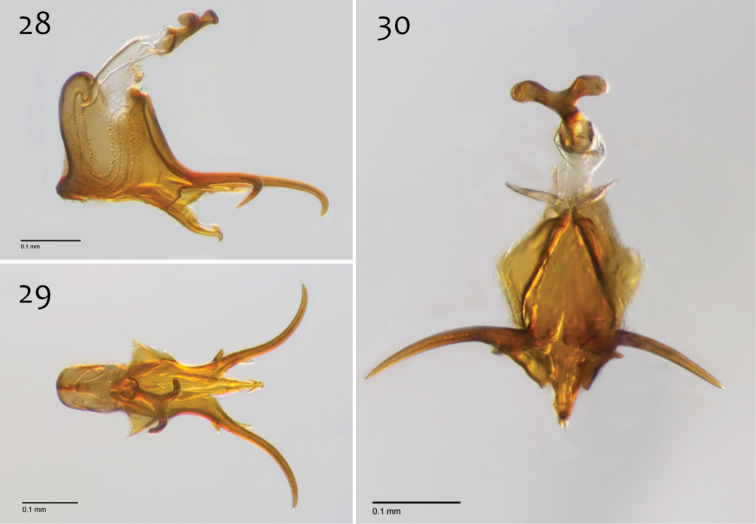
*Megophthalmidia lenimenta* sp. n., male aedeagus [paratype, # 13M317] **28** Lateral view **29** Dorsal view **30** Posterior view. Scale bar = 0.1 mm.

*Male genitalia* (Figs 22–30). Epandrium dorsal surface with clear medial depression, where setae are lacking; posterior margin narrowly emarginate at center (Fig. 24). Posterior processes of epandrium elongate, approx. 4–5× longer than narrowest width near base, separated at base by approx. 0.8× narrowest width of process, length of setae at base of epandrial processes 2–3× width of process, bare along most of length (Figs 22, 23). Gonocoxites as in Figs 25–27. Adeagal fork uneven; one tine a mere nub, the other, elongate, gently s-curved, and pointed outward (Fig. 29).

Female. Body length: 2.6–3.1, 2.9 mm (n=3). Antennal length 1.0–1.1, 1.1 mm (n=3). Wing length: 2.6–3.2, 3.0 mm (n=3).

*Coloration* (Fig. 21). Noticeably darker in color throughout body; abdominal segments 8–10 orange-brown to brown, brown along margins; cerci light brown to brown.

*Head and thorax*. Same as male, except palpomere 4 appx. length of palpomeres 1–3.

#### Etymology.

The species epithet “lenimenta” is an adjective, derived from the Latin word for remedy/melioration/reclamation. This name is given in thanks to the preservation efforts of Sylvia Mclaughlin and the University of California Donald and Sylvia Mclaughlin Reserve staff, including Cathy Koehler, and the Homestake Mining Company.

### Megophthalmidia mckibbeni sp. n.

http://zoobank.org/7EC54090-F153-4F18-BCFA-0C902ABCE7E3

http://species-id.net/wiki/Megophthalmidia_mckibbeni

**Figure 31.**
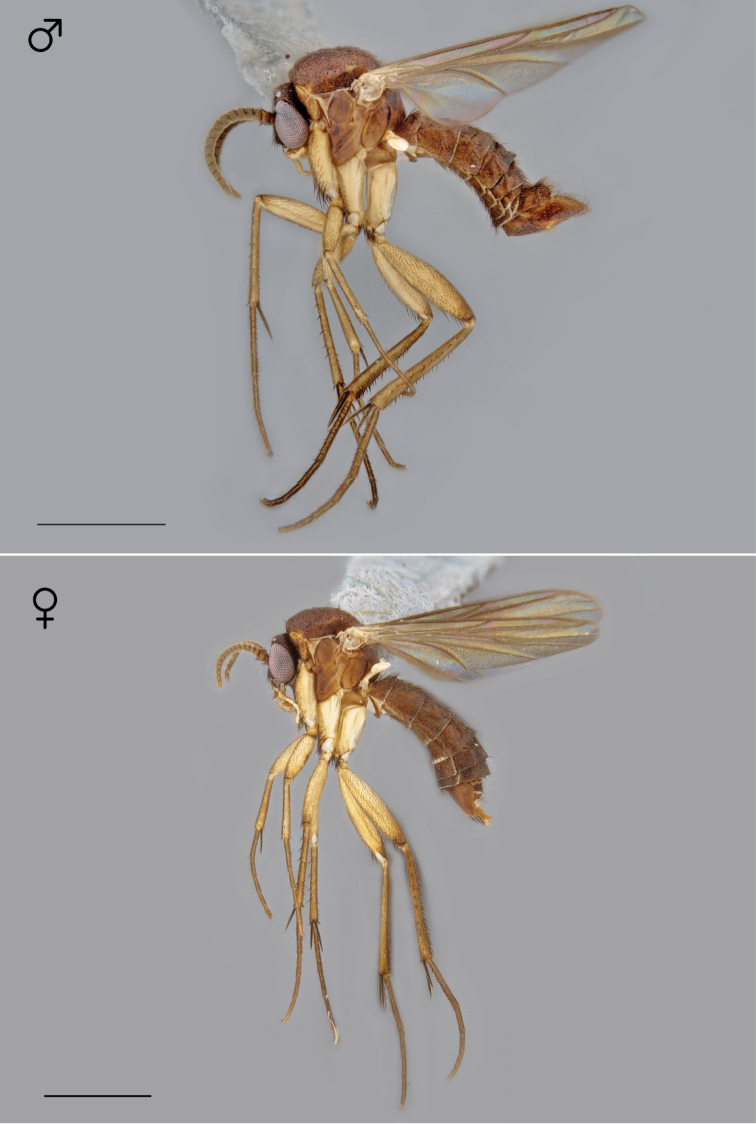
*Megophthalmidia mckibbeni* sp. n., habitus [holotype male above, # 12K727; female below, # 07Z047]. Scale bar = 1 mm.

Figs 31 [Fig F14] [Fig F15] –40

#### Type material.

Holotype: ♂, “USA: CA: Amador Co.: Indian Grinding Rock St. Pk., dry wash nr. S. Nature trail, MT#2, 38°25’ N, 120°38’ W’, 715masl, 10–29.vi.2007 P. Kerr & M. Hauser 07LOT315” / “HOLOTYPE 12K727, *Megophthalmidia mckibbeni* ♂, Kerr, 2014” [red label]. Deposited in CSCA, mounted on gray point, complete specimen (Fig. 31). Locality as in Fig. 104.

**Figures 32–34. F14:**
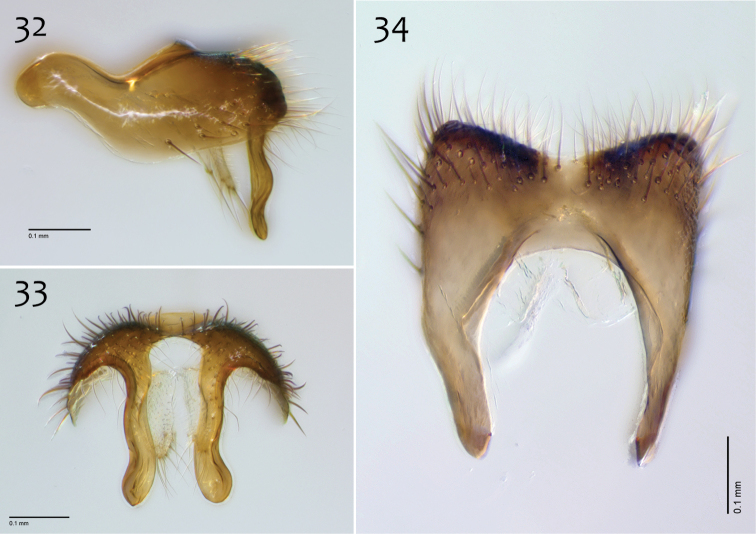
*Megophthalmidia mckibbeni* sp. n., male epandrium [paratype, # 12J954] **32** Lateral view **33** Posterior view **34** Dorsal view. Scale bar = 0.1 mm.

**Figures 35–37. F15:**
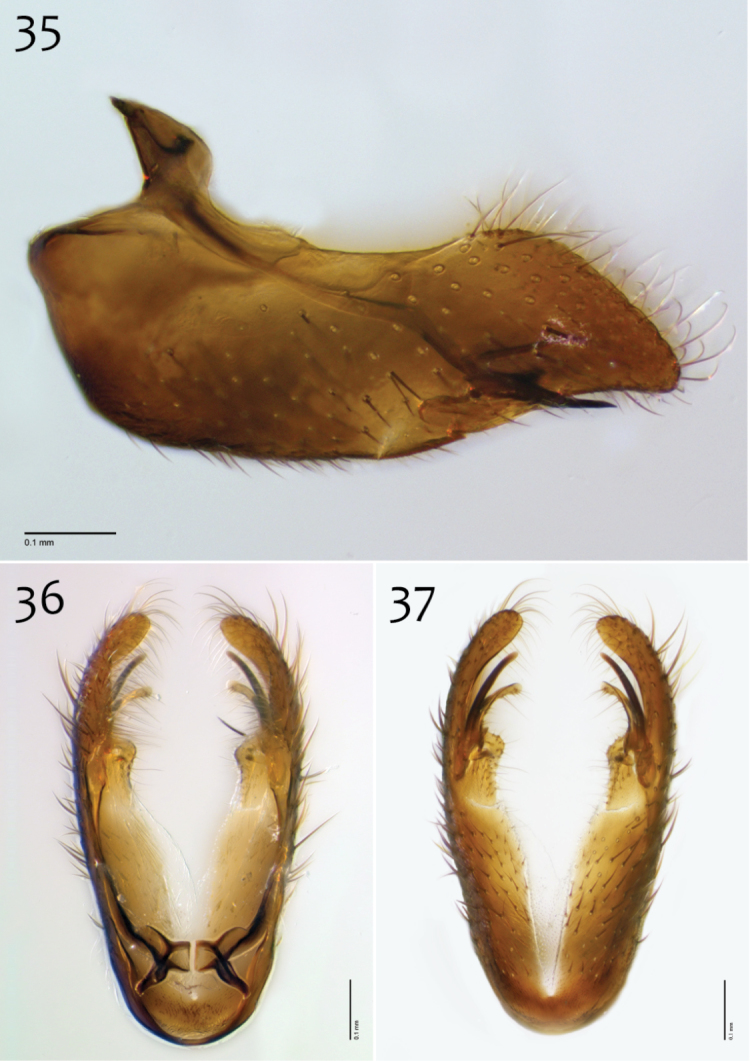
*Megophthalmidia mckibbeni* sp. n., male hypandrium [paratype, # 12J954] **35** Lateral view **36** Dorsal view **37** Ventral view. Scale bar = 0.1 mm.

**Figures 38–40.**
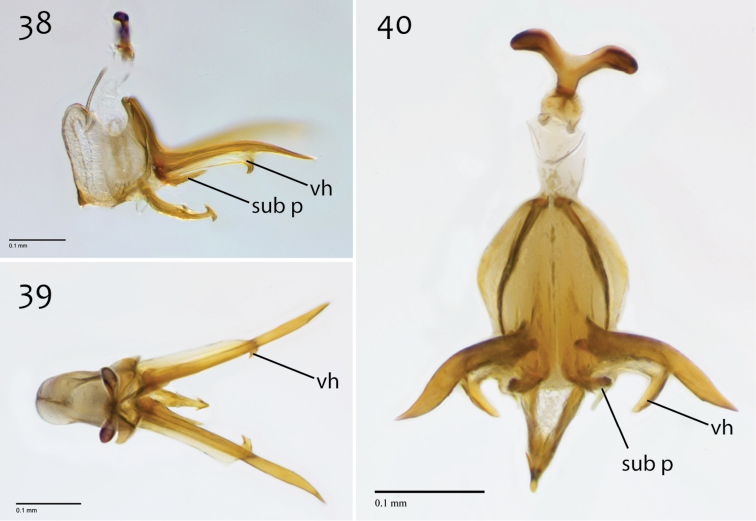
*Megophthalmidia mckibbeni* sp. n., male aedeagus [paratype, # 12J954] **38** Lateral view **39** Dorsal view **40** Posterior view. Scale bar = 0.1 mm. Abbreviations: **sub p** subtending process **vh** recurved ventral hook.

Paratypes (all bearing blue paratype labels): 3 ♂♂, 4 ♀♀, same locality as holotype [CSCA 2 ♀♀ including # 07Z047 (Fig. 31); LACM (♂, ♀); SBNM (♀)]; ♂, “USA: CA: Alpine Co.; Grover Hot Springs St. Pk., nr. Hoffman house, 38°41.997'N, 119°50.805'W; 1800masl, 8–22.vi.2006 PH Kerr & SL Winterton, MT#8 06LOT348” [CSCA]; ♂, “USA: CA: Amador Co.: Indian Grinding Rock SP, near campsites in gully, MT#1, 38°25.3’ N, 120°38.7 W, 715masl, 24.v–10.vi.2007 P. Kerr & P. Raggio, 07LOT095” [SBNM]; ♂, ♀, “USA: CA: Amador Co.: Indian Grinding Rock St. Pk., dry wash nr. S.Nature trail, MT#2, 38°25’ N, 120°38’ W’, 715masl, 24.v–10.vi.2007 P. Kerr & M. Hauser 07LOT096” [UAIC]; ♂, 3 ♀♀, “USA: CA: Amador Co.: Indian Grinding Rock St. Pk., dry wash nr. S.Nature trail, MT#2, 38.4216°N,-120.645°W 715masl, 15.v–18.vi.2008 P. Kerr CSCA08L596” [CSCA; ♂ with specimen # 13M404]; ♂, “USA: CA: Calaveras Co., Calaveras Big TreesSP, South Grove old fire rd., 38°14.9'N, 120°15.45'W ~1400masl, 8–26.vii.2005, A.R. Cline & S.D. Gaimari, 06LOT289” [CSCA]; ♂, “USA: CA: Sonoma Co., Annadel SP, 0.9mi from park lot, Richardson trail, 38°26.11'N, 122°36.67'W 220masl, 6m MT, 3–26.iv.2007 P. Kerr & S. Blank, 07LOT029”, [Locality Fig. 103; CSCA; specimen #13M281]; ♂, “USA: CA: Sonoma Co., Annadel SP, 0.9mi from park lot, Richardson trail, 38°26.11'N, 122°36.67'W 220masl, 6m MT#3, 17.v–7.vi.2007 P. Kerr & S. Blank 07LOT196” [CSCA]; ♂, “USA: CA: Tulare Co.: Whitaker Forest, E. Eshom Crk. Drainage, nr. tree#142, 36.7062N,-118.9319W, 1650masl, MT, 16.vii–12.viii.2010 P.H. Kerr CSCA10L286” [CSCA; specimen # 12J954, dissected (Figs 32–40)].

Additional material examined: **CALIFORNIA:** 6 ♂♂, 4 ♀♀, same locality as holotype [CSCA, **3**♂♂, ♀; SBNM, ♂, ♀; SBNM, ♂, ♀; UAIC, ♂, ♀]; ♂, “USA: CA: Alpine Co.; Grover Hot Springs St. Pk., nr. Hoffman house, 38°41.997'N, 119°50.805'W; 1800masl, 8–22.vi.2006 PH Kerr & SL Winterton, MT#8 06LOT348” [CSCA; specimen #12J962]; 12 ♂♂, 4 ♀♀, “USA: CA: Amador Co.: Indian Grinding Rock St. Pk., dry wash nr. S.Nature trail, MT#2, 38°25’ N, 120°38’ W’, 715masl, 24.v–10.vi.2007 P. Kerr & M. Hauser 07LOT096” [CSCA, 11 ♂♂, 3 ♀♀; SBNM, ♂, ♀; UAIC, ♂, ♀; Locality Fig. 104]; 23 ♂♂, 3 ♀♀, “USA: CA: Amador Co.: Indian Grinding Rock St. Pk., dry wash nr. S.Nature trail, MT#2, 38°25’ N, 120°38’ W’, 715masl, 10–29.vi.2007 P. Kerr & M. Hauser, 07LOT315” [CSCA; including specimen numbers 07Z050 (dissected ♂), 07Z069 (♂), and 07Z071 (♂)]; 8 ♂♂, 10 ♀♀, “USA: CA: Amador Co.: Indian Grinding Rock St. Pk., dry wash nr. S.Nature trail, MT#2, 38.4216°N, -120.645°W 715masl, 15.v–18.vi.2008 P. Kerr CSCA08L596” [CSCA, 7 ♂♂, 9 ♀♀; LACM ♂, ♀]; 4 ♂♂, “USA: CA: Calaveras Co., Calaveras Big TreesSP, South Grove old fire rd., 38°14.9'N, 120°15.45'W ~1400masl, 8–26.vii.2005, A.R. Cline & S.D. Gaimari, 06LOT289” [CSCA; one marked specimen #07Y096]; ♂, “USA: CA: Calaveras Co., Calaveras Big Trees SP, S. grove fire rd., nr. Beaver Creek, MT#1, 38°15.41'N, 120°15.25'W 1385masl, 22.v.-11.vi.2007 P.H. Kerr & A.R.Cline 07LOT086” [CSCA; specimen # 10F289, in alcohol]; ♂, “USA: CA: Calaveras Co., BigTreesSP, S. Grove, Sequoia tree #298 (Creek), canopy trap (153’), bottom bottle, 38.2415°N, 120.2554°W 1405masl, 6–27.vi.2009, P. Kerr & R. Frizzell CSCA09L410” [CSCA; specimen # 09D648, in alcohol]; ♂, “USA: CA: Calaveras Co., BigTreesSP, S. Grove, Sequoia tree #298 (Creek), canopy trap (153’), bottom bottle, 38.2415°N, 120.2554°W 1405masl, 27.vi–18.vii.2009, P. Kerr & R. Frizzell CSCA09L428” [CSCA; specimen # 09D639, in alcohol]; ♂, “Mill Valley, Marin Co. Cal., 11.VI.50 / H. B. Leech / Caught in cheesecloth trap” [CAS]; ♂, “U.S.A. CAL. Napa Co., N. side Howell Mt., 2 mi. NNE. Angwin, 1300 ft. H. B. Leech, 16.V.1975” [CAS]; 3 ♂♂, “U.S.A. CAL. Napa Co., N. side Howell Mt., 2 mi. NNE. Angwin, 1300 ft. H. B. Leech, VI.1978” [CAS]; ♂, “U.S.A. CAL. Napa Co., N. side Howell Mt., 2 mi. NNE. Angwin, 1300 ft. H. B. Leech, VII.1980” [CAS; dissected specimen #13M590]; ♂, “CALIF. Napa Co. Snell Valley, Stage Coach Canyon road, at Spanish Valley trail. 4–V 1980, Hugh B. Leech collector” [CAS]; ♂, ♀ “USA: CA: Nevada Co., Nevada City, Beckman St., canopy trap in pine, 39.2682°N, 121.0219°W, 800masl 10–28.v.2008 P. Kerr CSCA08L577” [CSCA; in alcohol]; 3 ♂♂, “USA: CA: Sonoma Co., Annadel SP, 0.9mi from park lot, Richardson trail, 38°26.11'N, 122°36.67'W 220masl 6m MT, 3–26.iv.2007 P. Kerr & S. Blank, 07LOT029” [Locality Fig. 103; CSCA]; ♂, “USA: CA: Sonoma Co., Annadel SP, 0.9mi from park lot, ravine near Warren Richardson trail, 38°26.11'N, 122°36.67'W, 220masl, 6m MT, 26.iv–17.v.2007 P. Kerr & S. Blank 07LOT049” [CSCA; specimen #07Y263]; 2 ♂♂, “USA: CA: Sonoma Co., Annadel SP, 0.9mi from park lot, Richardson trail, 38°26.11'N, 122°36.67'W 220masl, 6m MT#3, 17.v–7.vi.2007 P. Kerr & S. Blank 07LOT196” [CSCA]; 42 ♂♂, “U.S.A. CALIFORNIA: Tuolumne County, Basin Creek Campground, 1–VI–1963, P. H. Arnaud, Jr.” [CAS]; 4 ♂♂, “U.S.A. CALIFORNIA: Tuolumne County, Basin Creek Campground, 2–VI–1963, P. H. Arnaud, Jr.” [CAS]. **MEXICO:** ♂, “La Zanja, el 6800 ft., VI–16–1953, MEX: B. Calif., Sierra San Pedro Martir, P. H. Arnaud, Jr.” [CAS].

#### Diagnosis.

*Megophthalmidia mckibbeni* sp. n. is separated from its apparent closest relative, *Megophthalmidia occidentalis*, by the brown coloration of its thorax and abdomen (Fig. 31) and the shape of the aedeagal complex (Figs 38–40). It is distinguished from other Nearctic *Megophthalmidia* by the morphology of its male reproductive structures, particularly the aedeagal fork, which bears a short recurved ventral hook (Figs 38, 40), and the posterior process of the epandrium, whose apex is swollen (wider than midpoint width), broadly rounded, and curved abaxially (Fig. 33). For additional information, see diagnosis of *Megophthalmidia occidentalis*.

#### Description.

Male. Body length: 2.5–2.9, 2.7 [2.7] mm (n=10). Wing length: 2.5–2.9, 2.6 [2.7] mm (n=10).

*Coloration* (Fig. 31). Head dark brown; antennal scape, pedicel, and flagellomeres brown; face dark brown, clypeus and labrum brown; palps and labellum cream-colored to pale yellow (palpomere 2 usually slightly darker than others). Thorax brown to dark brown throughout; scutum setae brown. Coxae cream-colored to pale yellow, femora becoming gradually darker dorsoapically, tibiae light brown to brown (hind tibia darkest), tarsi brown; hind tibial comb yellowish, preceded by 0–3 (usually 3) dark brown setae. Wing hyaline without markings, wing veins brown; haltere stem and knob cream-colored to pale yellow. Abdominal segments concolorous brown to dark brown, except sternites 1–3 usually paler light brown to brown. Terminalia brown to dark brown.

*Head*. Ocelli slightly raised, median ocellus in line with anterior margin of lateral ocelli, median ocellus approx. 0.5× size as lateral ocelli; lateral ocellus located approx. 1–1.3× diameter of ocellus from eye margin, separated from median ocellus by approx. twice its own diameter. Eyes with microsetae, which are approximately as long as width of facet. Frons microtrichose, without setae, flattened. Antennal length 0.9–1.1, 1.0 [1.1] mm (n=10) (approx. 1× length of head and thorax). Face clearly longer than wide, setose; clypeus and labrum microtrichose, without setae. Palpus with four palpomeres; palpomere 1 triangular in shape, without setae; other palpomeres with golden brown to dark brown setae; palpomere 2 bearing small pocket of sensilla; palpomere 1 length shorter or subequal in length to palpomere 2; palpomere 3 length subequal to or longer than combined length of palpomeres 1 and 2; palpomere 4 length 0.75–1× combined lengths of palpomeres 1–3.

*Thorax*. Dorsum with evenly-distributed, short, appressed setae, bearing longer setae only along lateral and posterior margins. Antepronotum, proepisternum, and laterotergite bearing setae; remaining lateral thoracic sclerites bare. Costal wing vein extends beyond R_5_, one-half to approx. two-thirds distance between R_5_ and M_1_; R_1_ approximately the same length as r-m; cubital fork proximad of r-m base (as in *Megophthalmidia occidentalis*, Fig. 52); R_1_, M_1_, M_2_, CuA_1_, and CuA_2_ with setae on upper surface (lacking setae on M_1_ + M_2_). Wing veins A1 and CuP absent.

*Male genitalia* (Figs 32–40). Epandrium dorsal surface flat or nearly so, with or without setae medially, posterior broadly but shallowly emarginate at center (Fig. 34). Posterior processes of epandrium greater than 7× longer than wide, separated at base by approx. 2× width of process, length of setae at base of epandrial processes ~1× width of process; apex of posterior process swollen (wider than midpoint width), broadly rounded, and curved abaxially (Figs 33). Gonocoxites as in Figs 35–37. Aedeagal fork with short recurved hook, bearing subtending process approx. 1/2 length of base to tip of hook (Fig. 38).

Female. Body length: 2.1–3.0, 2.6 mm (n=7). Antennal length: 0.6–0.8, 0.7 mm (n=7). Wing length: 2.2–2.8, 2.6 mm (n=7).

*Coloration* (Fig. 31). Same as male; cerci light brown to brown.

*Head and thorax*. Same as male, except palpomere 4 appx. length of palpomeres 2–3 or slightly longer, antenna length shorter.

#### Etymology.

The species epithet “mckibbeni” is given to this species in honor of William Earnest “Bill” McKibben, noted author, environmental activist, and founder of 350.org. The magnificent diversity of life on our planet depends on a stable climate, which is now under grave threat. There are solutions, but they require the wisdom, persistence, and activism that Bill McKibben exemplifies.

### Megophthalmidia misericordia sp. n.

http://zoobank.org/F10D2F4C-08BC-4EA6-9994-7EC0F1F36BB4

http://species-id.net/wiki/Megophthalmidia_misericordia

**Figure 41.**
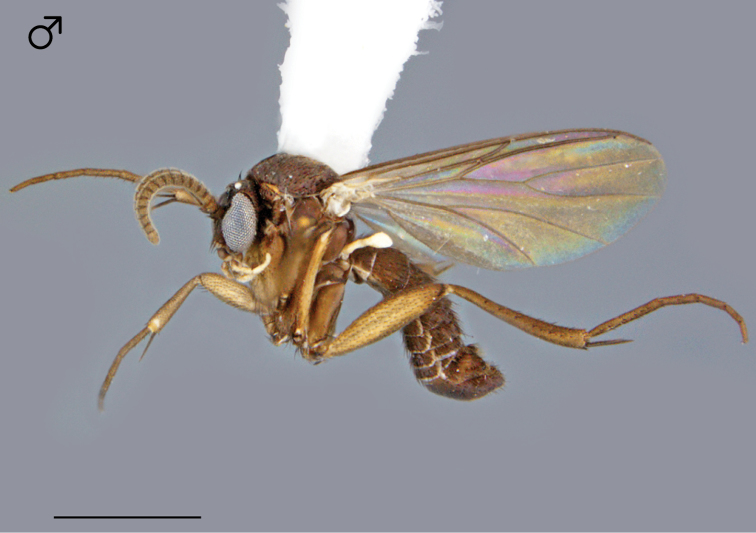
*Megophthalmidia misericordia* sp. n., habitus [holotype male, # 12J963; female unknown]. Scale bar = 1 mm.

Figs 41 [Fig F18] [Fig F19] –50

#### Type material.

Holotype: ♂, “USA: CA: Sonoma Co., Annadel SP, 0.9mi from park lot, Richardson trail, 38°26.11'N, 122°36.67'W 220masl 6m MT, 3–26.iv.2007 P. Kerr & S. Blank 07LOT029” / “HOLOTYPE 12J963, *Megophthalmidia misericordia* ♂, Kerr, 2014” [red label]. Deposited in CSCA, specimen glued directly to the pin, complete specimen (Fig. 41). See Fig. 103 for image of type locality.

**Figures 42–44. F18:**
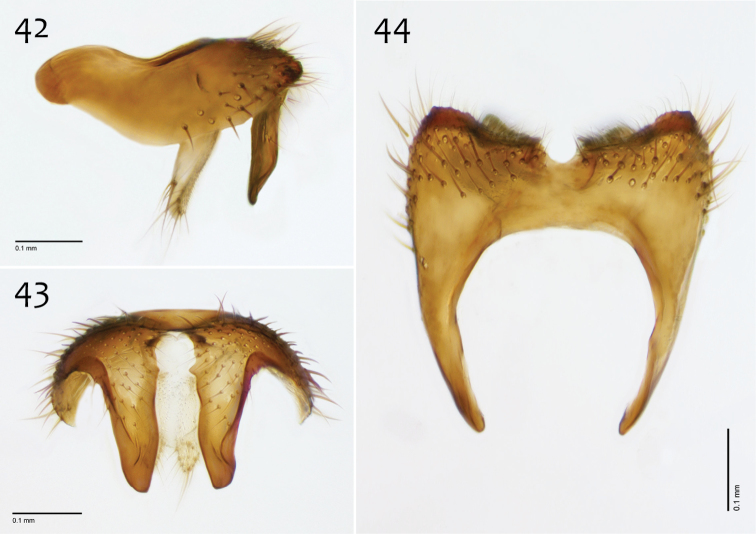
*Megophthalmidia misericordia* sp. n., male epandrium [paratype, # 12J985] **42** Lateral view **43** Posterior view **44** Dorsal view. Scale bar = 0.1 mm.

**Figures 45–47. F19:**
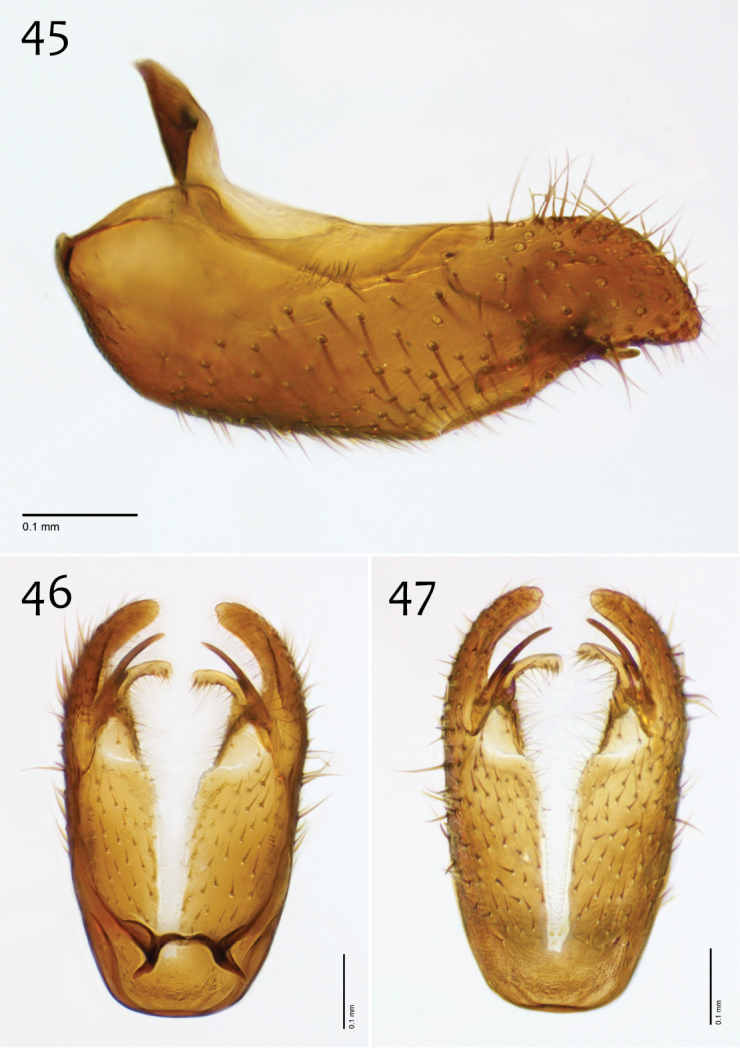
*Megophthalmidia misericordia* sp. n., male hypandrium [paratype, # 12J985] **45** Lateral view **46** Dorsal view **47** Ventral view. Scale bar = 0.1 mm.

**Figures 48–50.**
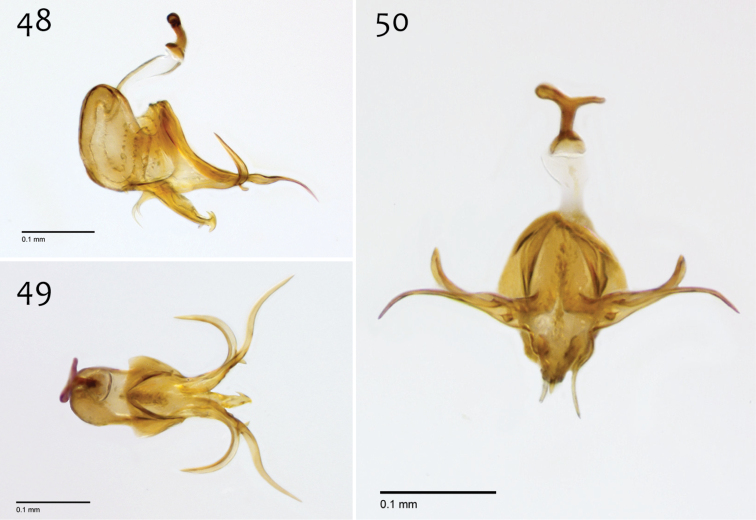
*Megophthalmidia misericordia* sp. n., male aedeagus [paratype, # 12J985] **48** Lateral view **49** Dorsal view **50** Posterior view. Scale bar = 0.1 mm.

Paratypes (all bearing blue paratype labels): ♂, “USA: CA: Sonoma Co., Annadel SP, 0.9mi from park lot, ravine near Warren Richardson trail, 38°26.11'N, 122°36.67'W, 220masl, 6m MT, 26.iv–17.v.2007 P. Kerr & S. Blank 07LOT049” [CSCA; specimen # 07Y264 (dissected)]; 9 ♂♂, “USA: CA: Sonoma Co., Annadel SP, 0.9mi from park lot, Richardson trail, 38°26.11'N, 122°36.67'W 220masl, 6m MT#3, 17.v–7.vi.2007 P. Kerr & S. Blank 07LOT196” [1 ♂ LACM; 8 ♂♂ CSCA including specimen numbers 12J984, 12J985 (Figs 42–50); four in alcohol, including # 12J958, # 12K739].

#### Diagnosis.

*Megophthalmidia misericordia* sp. n. may be confused with Nearctic congeners that also have a brown thorax and short and broad (stout) apical epandrial processes. Among these, it is most similar to *Megophthalmidia ignea* and *Megophthalmidia perignea* but may be distinguished from these species by having setose apical epandrial processes (not bare; Fig. 43) and narrow, elongate bifurcations of the aedeagal fork (Fig. 49). The narrow, elongate bifurcations of the aedeagal fork in *Megophthalmidia misericordia* are similar to those found in *Megophthalmidia browni*, but *Megophthalmidia misericordia* is distinguished by having stout apical epandrial processes (not elongate). *Megophthalmidia radiata* also has stout apical epandrial processes, but these are much broader at their base than in *Megophthalmidia misericordia*.

#### Description.

Male. Body length: 2.0–2.3, 2.1 [2.2] mm (n=6). Wing length: 2.0–2.2, 2.1 [2.1] mm (n=6).

*Coloration* (Fig. 41). Head dark brown; antennal scape dark brown, pedicel and flagellomeres brown; face dark brown, clypeus and labrum brown to dark brown; palps and labellum cream-colored to pale yellow (palpomeres 1 and 2 usually slightly darker than others, palpomere 2 with light patch where sensilla present). Thorax brown to dark brown throughout; scutum setae brown. Coxae lighter in color than thorax, brown; femora light brown to brown, becoming gradually darker dorsoapically; tibiae light brown to brown (hind tibia darkest), hind tibial comb dark brown, tarsi light brown to brown. Wing hyaline without markings, wing veins brown; haltere stem and knob white to cream-colored. Abdominal segments concolorous brown to dark brown. Terminalia brown.

*Head*. Ocelli slightly raised, median ocellus in line with anterior margin of lateral ocelli, median ocellus approx. 0.3–0.5× size of lateral ocelli; lateral ocellus located approx. 2× diameter of ocellus from eye margin, separated from median ocellus by approx. 2–2.2× its own diameter. Eyes with microsetae, which are approximately as long as width of facet. Frons microtrichose, without setae, flattened. Antennal length 0.7–0.8, 0.8 [0.7] mm (n=6). Face clearly longer than wide, setose; clypeus and labrum microtrichose, without setae. Palpus with four palpomeres; palpomere 1 barrel-shaped, without setae; other palpomeres with brown setae; palpomere 2 bearing small pocket of sensilla; palpomere 1 length longer than or subequal in length to palpomere 2; palpomere 3 length subequal to or slightly shorter than combined length of palpomeres 1 and 2; palpomere 4 length 0.8–1× combined lengths of palpomeres 1–3.

*Thorax*. Dorsum with evenly-distributed, short, appressed setae, bearing longer setae only along lateral and posterior margins. Antepronotum, proepisternum, and laterotergite bearing setae; remaining lateral thoracic sclerites bare. Costal wing vein extends beyond R_5_, approx. two-thirds distance between R_5_ and M_1_; R_1_ approximately the same length as r-m; cubital fork proximad of r-m base (as in *Megophthalmidia occidentalis*, Fig. 52); R_1_, M_1_, M_2_, CuA_1_, and CuA_2_ with setae on upper surface (lacking setae on M_1_ + M_2_). Wing veins A1 and CuP absent.

*Male genitalia* (Figs 42–50). Epandrium dorsal surface flat or nearly so, without setae medially, posterior margin narrowly emarginate at center (Fig. 44). Posterior processes of epandrium relatively wide, approx. 2.5× longer than wide, separated at base by approx. 0.5× width of process, length of setae at base of epandrial processes less than 0.5× width of process; apex of posterior process angled to dull point (Figs 43). Gonocoxites as in Figs 45–47. Aedeagal fork bifurcated into elongated tines of similar width; shorter tine broadly-curving upward and back, longer tine s-shaped (Figs 48–50).

Female unknown.

#### Etymology.

The species epithet “misericordia” is a noun in apposition, derived from the Latin word for pity/mercy. The species is known only from Annadel State Park, one of 70 California state parks that were scheduled to close in 2012 by California Governor Jerry Brown. Local support has kept this park in operation, but its economic foundation remains uncertain.

### Megophthalmidia occidentalis

Johannsen

http://species-id.net/wiki/Megophthalmidia_occidentalis

**Figure 51.**
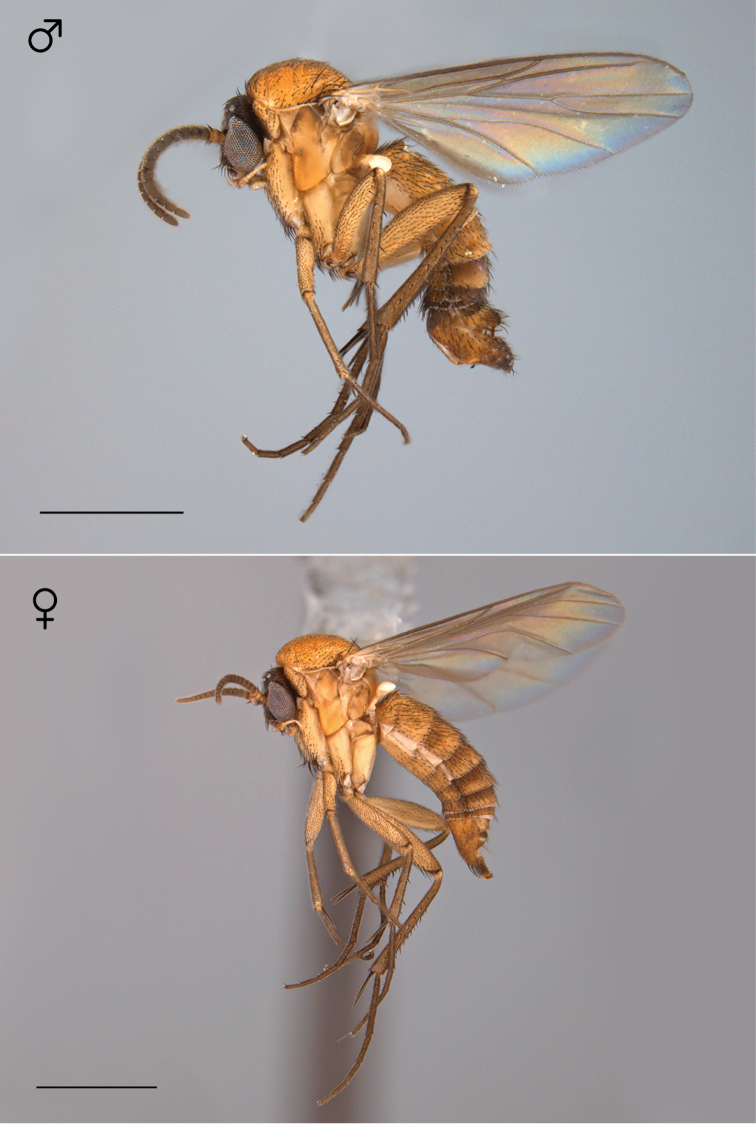
*Megophthalmidia occidentalis* Johannsen, habitus, fresh specimens [male above, # 07y273; female below, #07Z048]. Scale bar = 1 mm.

Figs 51 [Fig F22] [Fig F23] [Fig F24] –61

Megophthalmidia occidentalis Johannsen, 1909: 89

#### Type material examined.

♂, “Friday Harbor, Wash., July 6.05” / “OAJohannsen, Lot 114, Sub + slide, Cornell U.” / “♂ HOLOTYPE *Megophthalmidia occidentalis* Johannsen” [white label with edge colored red] / “HOLOTYPE Cornell U. No. 1999” [red label]. Specimen double-mounted. Left wing dissected and slide-mounted, marked “HOLOTYPE Cornell U., No. 1999”; male genitalia dissected, ethanol-preserved, in glass microvial within stoppered glass vial marked “MYCETOPHILIDAE, O.A. Johannsen, LOT 114, Cornell U., HOLOTYPE No. 1999.” [CUIC].

**Figure 52. F22:**
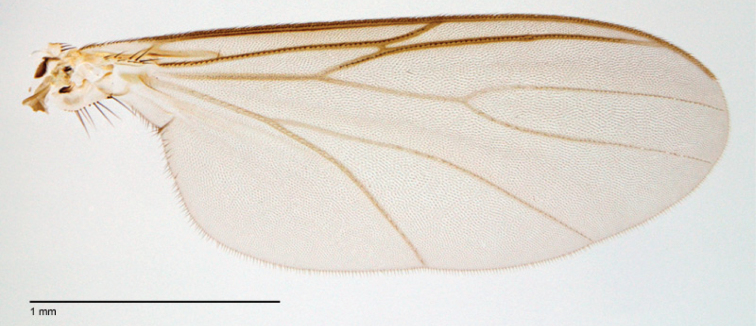
*Megophthalmidia occidentalis* Johannsen, wing [male, # 13M284]. Scale bar = 1 mm.

**Figures 53–55. F23:**
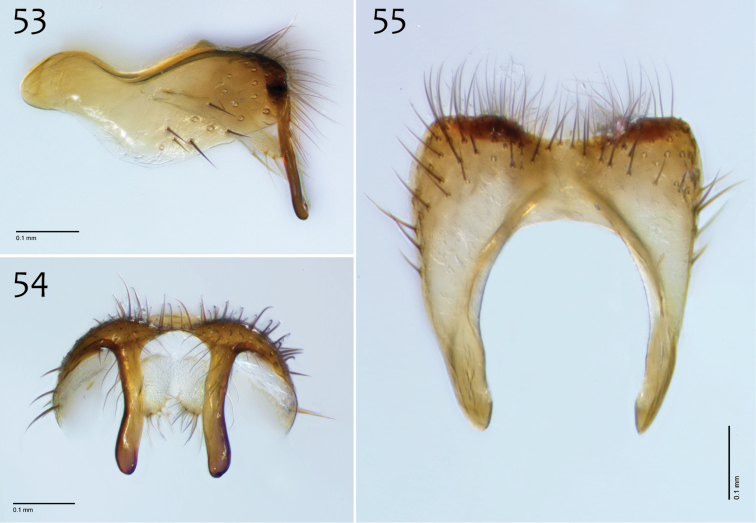
*Megophthalmidia occidentalis* Johannsen, male epandrium [# 12J955] **53** Lateral view **54** Posterior view **55** Dorsal view. Scale bar = 0.1 mm.

**Figures 56–58. F24:**
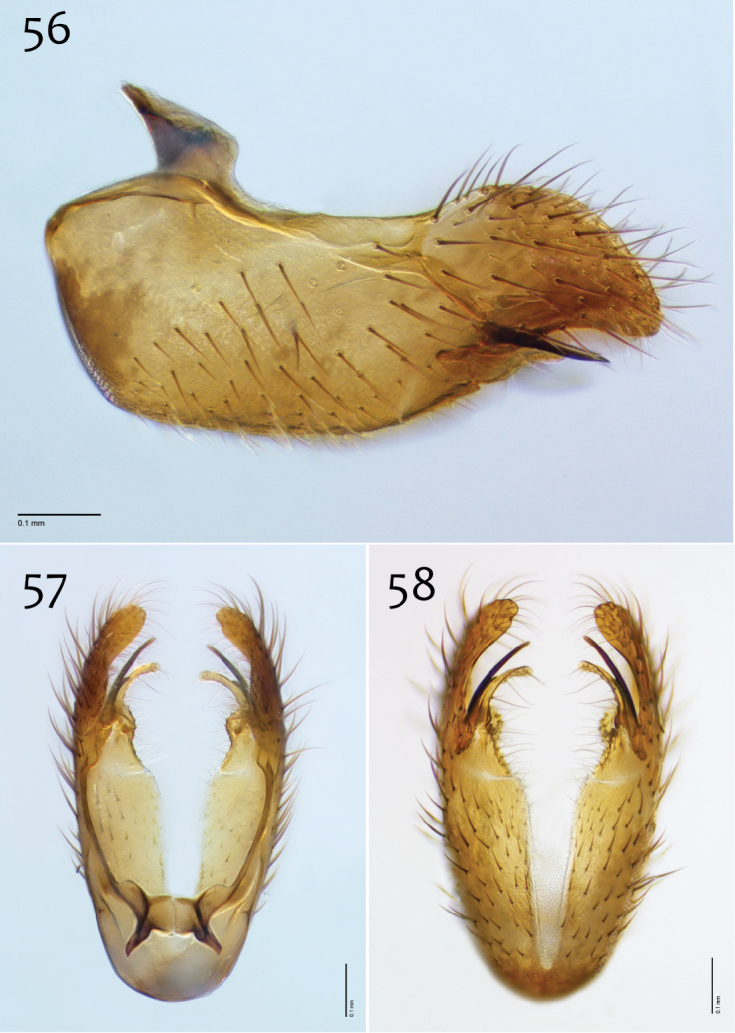
*Megophthalmidia occidentalis* Johannsen, male hypandrium [# 09D712] **56** Lateral view **57** Dorsal view **58** Ventral view. Scale bar = 0.1 mm.

**Figures 59–61.**
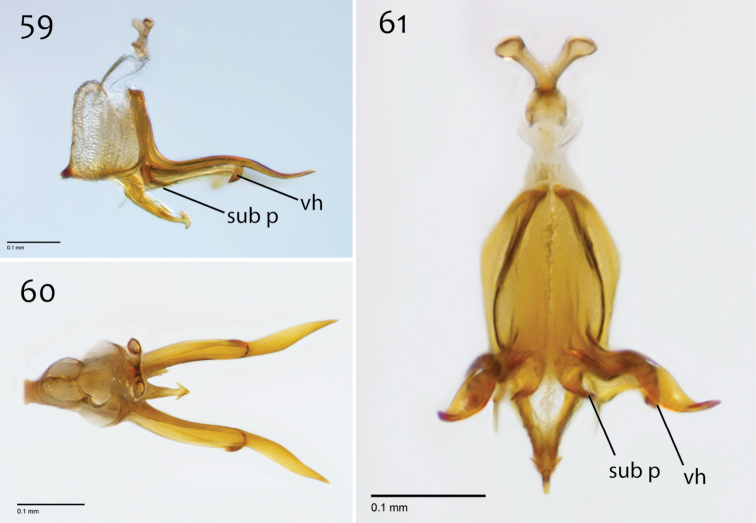
*Megophthalmidia occidentalis* Johannsen, male aedeagus [# 12J955] **59** Lateral view **60** Dorsal view **61** Posterior view. Scale bar = 0.1 mm. Abbreviations: **sub p** subtending process **vh** recurved ventral hook.

Additional material examined: **CALIFORNIA:** 2 ♂♂, 3 ♀♀, “USA: CA: Alpine Co; GroverHotSprings SP, nr. Hoffman house, 38°41.997'N, 119°50.805'W, 1800masl, 14.viii–3.ix.2006 PH Kerr & M Hoffman 06LOT476” [CSCA]; 4 ♂♂, 4 ♀♀, “USA: CA: Amador Co.: Indian Grinding Rock St. Pk., dry wash nr. S.Nature trail, MT#2, 38°25’ N, 120°38’ W’, 715masl, 24.v–10.vi.2007 P. Kerr & M. Hauser 07LOT096” [CSCA; locality Fig. 104; specimen numbers 07Y273 (♂, Fig. 51), 07Y274 (♂, dissected), and # 07Z068 (♀)]; ♂, “USA: CA: Amador Co.: Indian Grinding Rock St. Pk., firebreak nr. envtl camp, MT#3, 38°25.7’ N, 120°38.6’ W’, 715masl, 24.v–10.vi.2007 P. Kerr & M. Hauser 07LOT097” [CSCA]; 26 ♂♂, 46 ♀♀, “USA: CA: Amador Co.: Indian Grinding Rock St. Pk., dry wash nr. S.Nature trail, MT#2, 38°25’ N, 120°38’ W’, 715masl, 10–29.vi.2007 P. Kerr & M. Hauser, 07LOT315” [CSCA, 25 ♂♂, 45 ♀♀, including specimen numbers 07Z048 (♀, Fig. 51) and 07Z049 (♀, dissected); UAIC, ♂, ♀]; 6 ♂, 13 ♀♀, “USA: CA: Amador Co.: Indian Grinding Rock St. Pk., dry wash nr. S.Nature trail, MT#2, 38.4216°N,-120.645°W 715masl, 15.v–18.vi.2008 P. Kerr CSCA08L596” [CSCA, 4 ♂, 11 ♀♀; LACM, ♂, ♀; SBNM, ♂, ♀]; 6 ♂♂, 2 ♀♀, “USA: CA: Amador Co., Indian Grinding Rock SHP, 25.vi.2008, P. Kerr CSCA09L616” [CSCA; in alcohol]; 13 ♂♂, 16 ♀♀, “USA: CA: Calaveras Co., Calaveras Big TreesSP, South Grove old fire rd., 38°14.9'N, 120°15.45'W ~1400masl, 8–26.vii.2005, A.R. Cline & S.D. Gaimari, 06LOT289” [CSCA, 10 ♂♂, 13 ♀♀; LACM, ♂, ♀; SBNM, ♂, ♀; UAIC, ♂, ♀;]; ♂, “USA: CA: Calaveras Co., BigTreesSP, S. Grove, Sequoia tree #298 (Creek), canopy trap (153’), 38.2415°N, 120.2554°W 1405masl, 27.vi–18.vii.2009, P. Kerr & R. Frizzell CSCA09L428” [CSCA; in alcohol, specimen # 09D638]; ♂, “USA: CA: Calaveras Co., BigTreesSP, S. Grove, Sequoia tree #282 (Stellar), canopy trap (129’), top bottle, 38.2407°N, 120.2546°W 1425masl, 27.vi–18.vii.2009, P. Kerr & R. Frizzell CSCA09L433” [CSCA; specimen # 09D656, in alcohol]; ♂, “USA: CA: Calaveras Co., BigTreesSP, S. Grove, Sequoia tree #282 (Stellar), canopy trap (129’), bottom bottle, 38.2407°N, 120.2546°W 1425masl, 27.vi–18.vii.2009, P. Kerr & R. Frizzell CSCA09L434” [CSCA; specimen # 09D656, in alcohol]; 4♀♀, “USA: CA: Calaveras Co., BigTreesSP, S. Grove, Sequoia tree #282 (Stellar), canopy trap nr. ground, top bottle, 38.2407°N, 120.2546°W 1425masl, 27.vi–18.vii.2009, P. Kerr & R. Frizzell CSCA09L435” [CSCA; in alcohol]; 2 ♂♂, “USA: CA: Calaveras Co., BigTreesSP, S. Grove, Sequoia tree #282 (Stellar), canopy trap nr. ground, bottom bottle, 38.2407°N, 120.2546°W 1425masl, 27.vi–18.vii.2009, P. Kerr & R. Frizzell CSCA09L436” [CSCA; specimen numbers 09D665, 09D666, both in alcohol]; ♂, “USA: CA: Calaveras Co., BigTreesSP, S. Grove, Sequoia tree #317 (Neighbor), canopy trap (4’), top bottle, 38.2406°N, 120.2563°W 1410masl, 27.vi–18.vii.2009 P. Kerr & R. Frizzell CSCA09L441” [CSCA; specimen # 09D718, in alcohol]; ♂, “USA: CA: Del Norte Co, SixRiversNF, ForestRd17N05, 2miSE Rt.199, 41.8737°N, 123.8135°W, 620masl, 3.vi–24.vii.2009 P. Kerr & O. Lonsdale, 2m MT, CSCA09L524” [CSCA; Figs 56–58]; 2 ♂♂, “CALIF. Madera Co., Meadow near locked gate, road to Mark Mine, SE. slope of Green Mt. 7600 ft., 20.VIII.71 H. Leech” [CAS]; 6 ♂♂, “CAL. Mono Co., Alt. 7200 ft, Leavitt Meadow / 12–VIII–1963, flight trap, H. B. Leech” [CAS]; ♂, “McBride Spgs., Mt. Shasta, CALIF., 4800’ 22.VII.1965, Malaise trap” [CNC]; ♂, “Loop Trail nr, Phillipsville, Alt Hwy 101 CAL, 5-VII-1968, B. V. Peterson” [CNC]; ♂, “U.S.A.: CALIFORNIA: Siskiyou County, McBride Springs, 8–VIII–1967 1524m., Paul H. Arnaud, Jr.” [CAS]; ♂, “U.S.A.: CALIFORNIA: Siskiyou County, Elk Creek, ca. 6 km. E. McCloud, 29–VII–1974, Paul H. Arnaud, Jr., Calif. Acad. Sci. Coll.” [CAS]; ♂, “U.S.A.: CALIFORNIA: Siskiyou County, Big Flat Public Camp, South Fork Salmon River, 20–VIII–1980, 1510m, Paul H. Arnaud, Jr.” [CAS]; 10 ♂♂, 2 ♀♀, “USA: CA: Sonoma Co., Annadel SP, 0.9mi from park lot, Richardson trail, 38°26.11'N, 122°36.67'W 220masl, 6m MT#3, 17.v–7.vi.2007 P. Kerr & S. Blank 07LOT196” [CSCA; including # 13M284 (♂, Fig. 52), 7 males in alcohol]; ♂, “USA: CA: Sonoma Co., Annadel SP, swept from exposed roots overhanging dry creekbed, 38°26.11'N, 122°36.67'W 220masl 7.vi.2007 P. Kerr 07LOT094” [CSCA]; ♂, “CAL. Trinity Co., S. Fork Van Horn, creek 2 mi. from, mouth at upper, Mad river. Alt. 3000’ 9.VIII.70 / moss-edged rock pools in running stream, open area. Collector Hugh B. Leech” [CAS]; ♂, “USA: CA: Tulare Co.: Whitaker Forest, E. Eshom Crk. Drainage, nr. tree #142, 36.7062N, 118.9319W, 1650masl, MT, 3.vi–16.vii.2010 P.H. Kerr CSCA10L174” [CSCA; specimen #12J131, in alcohol]; 3 ♂♂, “USA: CA: Tulare Co.: Whitaker Forest, E. Eshom Crk. Drainage, nr. tree #142, 36.7062N, 118.9319W, 1650masl, MT, 16.vii–12.viii.2010 P.H. Kerr CSCA10L286” [CSCA; # 12J955 (dissected, Figs 53–55, 59–61); 2 ♂♂ in alcohol]. **IDAHO:** ♂, “USA: IDAHO: Bonner County, Sandpoint KOA, 0.4 km W. Sagle, 18–VII-1974, Paul H. Arnaud, Jr., Calif. Acad. Sci. Coll.” [CAS (dissected)]. **NEVADA:** ♂, “Clear Creek Cpgd., Ormsby Co., NEV., 6500’ 27.VII.1968, D. D. Munroe” [CNC]. **OREGON:** 12 ♂♂, “USA: ORE.: Deschutes Co., DeschutesRiver, 1 mi. SW. Pringle Falls 31–VII-1970, Paul H. Arnaud, Jr., 4250’ Flight Trap” [CAS]; ♂, “USA: OREGON: Crook County, Cougar Campground, Marks Creek, 24–VII–1974, Paul H. Arnaud, Jr., Calif. Acad. Sci. Coll.” [CAS]; ♂, “USA: OREGON: Jackson County, Union Creek Campground, Rogue River 17.3 km N. Prospect, 975m / 24–VII–1974, Paul H. Arnaud, Jr., Calif. Acad. Sci. Coll.” [CAS]. **WYOMING:** ♂, “U.S.A.: WYOMING:, Lincoln County, between Grover and, Thayne 16–VIII–1981, Paul H. Arnaud, Jr” [CAS]. **CANADA (B.C.**): ♂, “12km. NE Pemberton, B.C., 28-VII-1990, A. Borkent CD1236” [CNC]; ♂, “No. 62-1256-04, Date 8.VII.62, F.I.S.1962 / light trap, Langford, B.C.” [CNC]; ♂, “CANADA: BC: Nanaimo, 49.13°N, 123.97°W, 4–6 June 2004, B. Brown, Malaise 06LOT435” [CSCA; specimen # 13N141].

#### Diagnosis.

*Megophthalmidia occidentalis* is most easily separated from *Megophthalmidia mckibbeni* sp. n. and all other Nearctic congeners by its yellow body that contrasts its dark brown head (Fig. 51). This species is most similar to *Megophthalmidia mckibbeni* in the morphology of its male reproductive structures, particularly the aedeagus, which features a pair of long lateral processes (= “aedeagal fork”, Fig. 60) that bear a short recurved ventral hook (Fig. 59). This feature distinguishes these two species from all other *Megophthalmidia* in North America. Independent of body coloration, *Megophthalmidia occidentalis* may be distinguished from *Megophthalmidia mckibbeni* by having the epandrium posterior margin emarginate medially only (Fig. 55; not entire margin angled inward, toward center), aedeagus bearing lateral processes that have s-curvature (tip pointing upward) in lateral view (Fig. 59) and are subtended by a short process that extends about 1/3 length of lateral process before hook (Fig. 59; in *Megophthalmidia mckibbeni*, this subtending process is clearly longer (Fig. 38)). In the dorsal view, the aedeagal lateral processes of *Megophthalmidia occidentalis* are thicker and exhibit noticeable bends (Fig. 60), as opposed to being largely straight (as in *Megophthalmidia mckibbeni*, Fig. 39).

#### Description.

Male. Body length: 2.3–3.0, 2.7 [2.9] mm (n=10). Wing length: 2.2–2.7, 2.5 [2.6] mm (n=10).

*Coloration* (Fig. 51). Head dark brown; antennal scape and pedicel pale cream-colored to pale yellow, flagellomeres brown; face and clypeus light brown to brown; palps and labellum cream-colored to pale yellow. Thorax pale yellow to yellow throughout; scutum setae brown. Legs cream-colored to pale yellow becoming gradually darker apically, to light brown at tarsi; hind tibial comb yellowish, preceded by 0–3 (usually 3) dark brown setae. Wing hyaline without markings, wing veins yellowish brown; haltere stem and knob pale yellow. Anterior abdominal segments yellow to yellowish brown, posterior segments darkening successively; all abdominal segments bear dark brown setae. Terminalia pale yellow to light brown at base, gradually becoming darker brown apically.

*Head*. Ocelli slightly raised, median ocellus in line with anterior margin of lateral ocelli, median ocellus approx. 0.5× size as lateral ocelli; lateral ocellus located approx. 1–1.3× diameter of ocellus from eye margin, separated from median ocellus by approx. twice its own diameter. Eyes with microsetae, which are approximately as long as width of facet. Frons microtrichose, without setae, flattened. Antennal length 0.8–1.1, 1.0 [1.0] mm (n=10) (approx. 1× length of head and thorax). Face clearly longer than wide, setose; clypeus and labrum microtrichose, without setae. Palpus with four palpomeres; palpomere 1 without setae, other palpomeres with golden brown to dark brown setae, palpomere 2 bearing inconspicuous, small pocket of sensilla; palpomeres 1 and 2 subequal in length, palpomere 3 length subequal to or shorter than combined length of palpomeres 1 and 2, palpomere 4 length 1–1.25× combined lengths of palpomeres 1–3. In female, palpomere 4 appx. length of palpomeres 2–3.

*Thorax*. Dorsum with evenly-distributed, short, appressed setae, bearing longer setae only along lateral and posterior margins. Antepronotum, proepisternum, and laterotergite bearing setae; remaining lateral thoracic sclerites bare. Costal wing vein extends beyond R_5_, one-half to approx. two-thirds distance between R_5_ and M_1_; R_1_ approximately the same length as r-m; cubital fork proximad of r-m base (Fig. 52); R_1_, M_1_, M_2_, CuA_1_, and CuA_2_ with setae on upper surface (lacking setae on M_1_ + M_2_). Wing veins A1 and CuP absent.

*Male genitalia* (Figs 53–61). Epandrium dorsal surface flat or nearly so, with or without setae medially, posterior broadly but shallowly emarginate at center (Fig. 55). Posterior processes of epandrium greater than 7× longer than wide, separated at base by approx. 3× width of process, length of setae at base of epandrial processes ~2× width of process (Figs 54). Gonocoxites as in Figs 56–58. Aedeagal fork with short recurved hook, bearing subtending process approx. 1/3 length of base to tip of hook (Fig. 59).

Female. Body length: 2.4–3.0, 2.7 mm (n=10). Antennal length: 0.6–0.8, 0.7 mm (n=10). Wing length: 2.4–2.7, 2.6 mm (n=10).

*Coloration* (Fig. 51). Same as male, except generally lighter at apex of abdomen.

*Head and thorax*. Same as male, except antenna length shorter; 0.6–0.8, 0.7 mm (n=10).

#### Discussion.

Where sympatric, *Megophthalmidia occidentalis* and *Megophthalmidia mckibbeni* demonstrate clear color differences of both males and females that make distinguishing them in the field routine (Figs 31, 51). Interestingly, the male genitalia of these two species are remarkably similar however – to the point where additional study was necessary to make sure that they aren’t simply color morphs of the same species. This wouldn’t be expected, given that both sexes of each type are found in sympatry, but it was still worth considering. As a test in a ‘double-blind’ format, I examined dozens of dissected and disassociated male genitalia from both *Megophthalmidia mckibbeni* and *Megophthalmidia occidentalis* throughout their respective geographic ranges, to see if differences in the structure of the male genitalia are consistent with the more obvious, non-genitalic differences between these species, such as body color. It was found that the genitalia of these two species do exhibit consistent morphological differences, in addition to differences of body color, and this test corroborated a separate species hypothesis for each (i.e., they can be distinguished reliably by their genitalia alone). If specimens are not critically-point dried, however, specimens of *Megophthalmidia occidentalis* tend to darken to an orange-brown color and in Southern California, I have seen a male *Megophthalmidia occidentalis* that retains a similar body color to *Megophthalmidia mckibbeni* [#12K748], so specific morphology of the male terminalia remains the more reliable arbiter for proper species recognition. The original holotype of *Megophthalmidia occidentalis* has darkened somewhat, but is consistent with Fig. 51.

### Megophthalmidia perignea sp. n.

http://zoobank.org/B80ABEC9-7043-4E7C-8273-EF6C2176B7C8

http://species-id.net/wiki/Megophthalmidia_perignea

**Figure 62.**
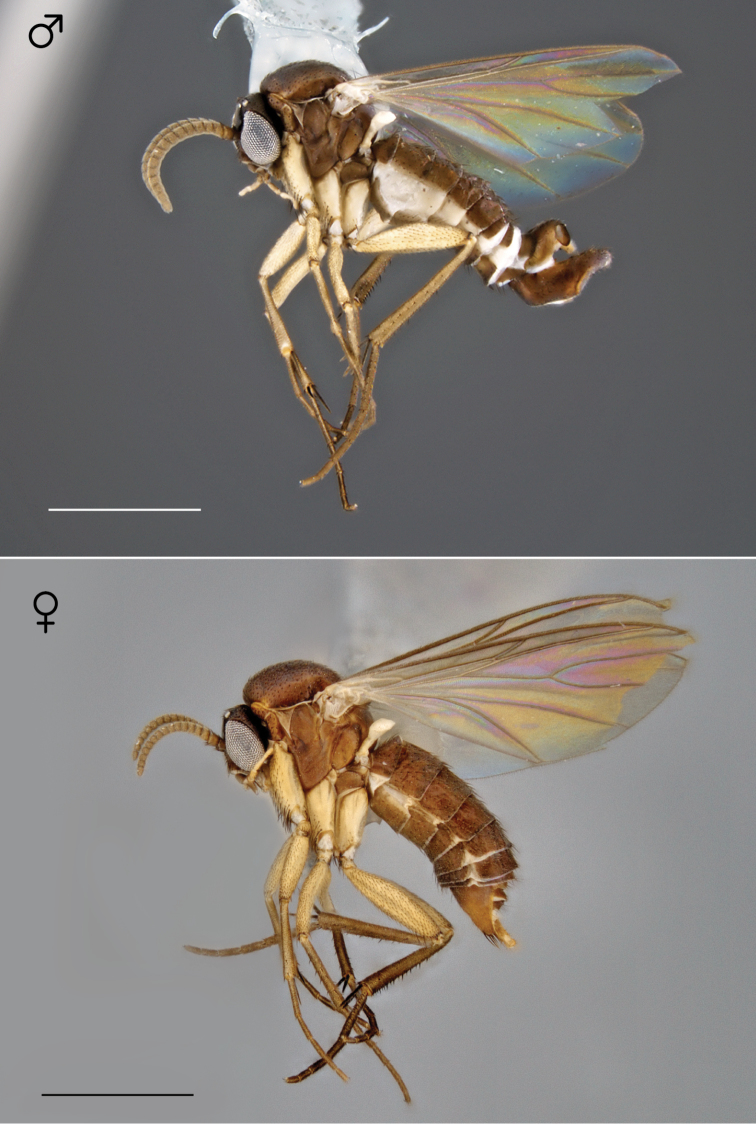
*Megophthalmidia perignea* sp. n., habitus [holotype male above, # 13N210; female below, # 13N394]. Scale bar = 1 mm.

Figs 62 [Fig F27] [Fig F28] –71

#### Type material.

Holotype: ♂, “USA: ARIZONA: Mohave Co; 11km, ESE Kingman; Malaise nr Hualapai, Mt. Recreational Park; 5–15.vi.2012, ME Irwin; 1740m; 35°06.68'N, 113°54.14'W CSCA13L225” / “HOLOTYPE 13N210, *Megophthalmidia perignea* ♂, Kerr, 2014” [red label]. Deposited in CSCA, mounted on gray point, entire specimen in excellent condition (Fig. 62), missing part of left wing.

**Figures 63–65. F27:**
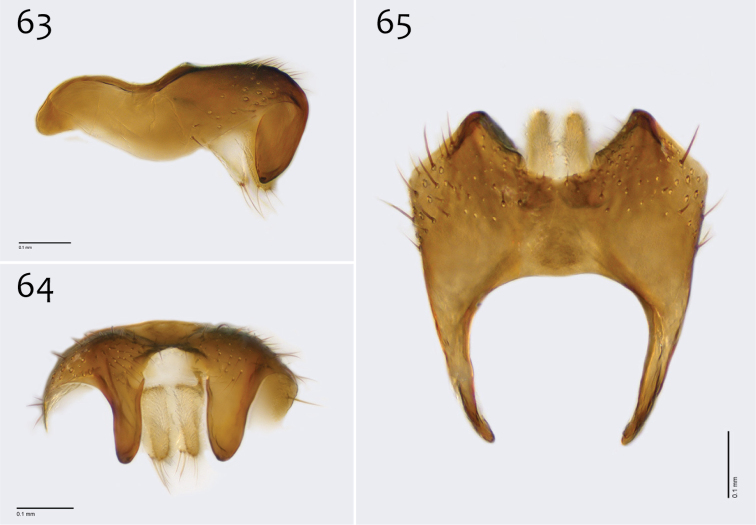
*Megophthalmidia perignea* sp. n., male epandrium [paratype, # 13M587] **63** Lateral view **64** Posterior view **65** Dorsal view. Scale bar = 0.1 mm.

**Figures 66–68. F28:**
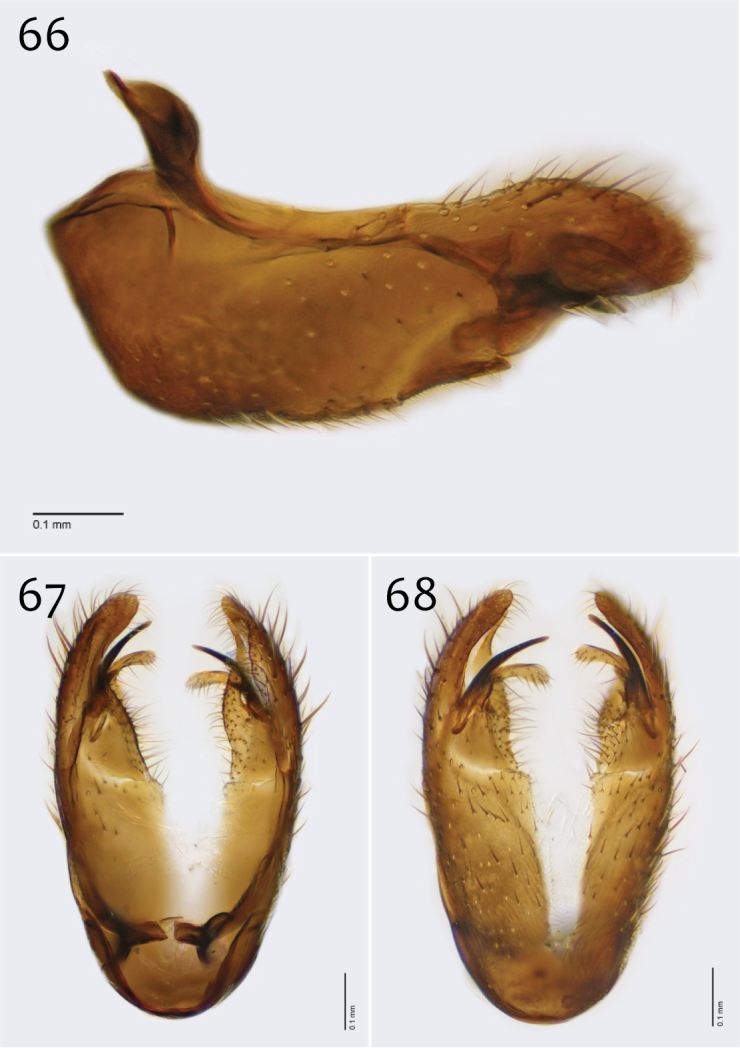
*Megophthalmidia perignea* sp. n., male hypandrium [paratype, # 12J587] **66** Lateral view **67** Dorsal view **68** Ventral view. Scale bar = 0.1 mm.

**Figures 69–71.**
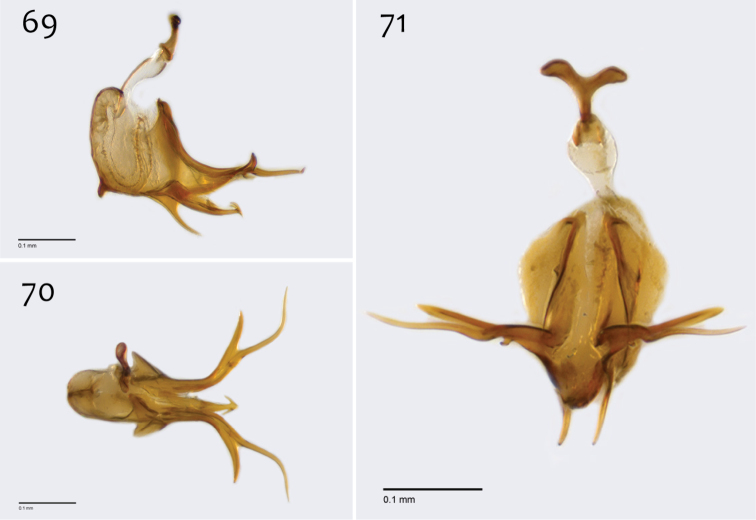
*Megophthalmidia perignea* sp. n., male aedeagus [paratype, # 13M587] **69** Lateral view **70** Dorsal view **71** Posterior view. Scale bar = 0.1 mm.

Paratypes (all bearing a blue paratype label): 4 ♂♂, 3 ♀♀, same locality as holotype / [CSCA; specimen numbers 13M587 (dissected ♂, Figs 63–71), 13M569 (♂), 13M659 (♂, in alcohol), 13N390 (♂), 13N391 (♂),13N392 (♀), 13N393 (♀), 13N394 (♀, Fig. 62)]; ♂, “USA: ARIZONA, Gila Co; 21km S Globe; Malaise in Oak-Juniper, hillside thicket; 15–20.v.2013, ME Irwin; 1480m; 33°14.12'N, 110°46.92'W CSCA13L218” [CSCA; specimen # 13M544]; ♂, ♀, “USA: ARIZONA, Gila Co; 21km S Globe; Malaise in Oak-Juniper, hillside thicket; 20–25.v.2013, ME Irwin; 1480m; 33°14.12'N, 110°46.92'W CSCA13L222” [UAIC; specimen numbers 13M556 (♂), 13N389 (♀)].

#### Diagnosis.

Like its putative sister taxon, *Megophthalmidia ignea*, *Megophthalmidia perignea* sp. n. is superficially similar to *Megophthalmidia browni* and *Megophthalmidia mckibbeni* sp. n. because of its brown thorax and contrasting cream-colored tibia, but may be separated from these taxa by its shortened and bare apical epandrial processes (Figs 63, 64). *Megophthalmidia perignea* is similar to *Megophthalmidia ignea* in nearly all aspects except in relatively subtle features of the male genitalia. At its base, the short aedeagal tine of *Megophthalmidia perignea* has approximately the diameter as the long aedeagal tine. Also, in *Megophthalmidia perignea*, the apical epandrial processes are shorter, wider, and turned inward more than in *Megophthalmidia ignea*.

#### Description.

Male. Body length: 2.6–2.9, 2.8 [2.9] mm (n=6). Wing length: 2.5–2.9, 2.7 [2.5] mm (n=7).

*Coloration* (Fig. 62). Head dark brown; antennal scape, pedicel, and flagellomeres brown; face dark brown, clypeus and labrum brown to dark brown; palps and labellum cream-colored to pale yellow (palpomeres 1–3 usually slightly darker than others, palpomere 2 with light patch where sensilla present). Thorax brown to dark brown throughout, except at the anterolateral margin of the dorsum and dorsal apronotal area, where it may be narrowly cream-colored or pale yellow; scutum setae golden brown to dark brown. Coxae clearly lighter in color than thorax, cream-colored to pale yellow; femora cream-colored throughout, dark brown at apical margin; tibiae and tarsi cream-colored to pale yellow, with densely-arranged dark brown setae; hind tibial comb yellowish, preceded by 0–3 (usually 3) dark brown setae. Wing hyaline without markings, wing veins brown; haltere stem and knob white to cream-colored. Abdominal segments concolorous brown. Terminalia light brown to brown.

*Head*. Ocelli slightly raised, median ocellus in line with anterior margin of lateral ocelli, median ocellus approx. 0.3×–0.5× size of lateral ocelli; lateral ocellus located approx. 1.8× diameter of ocellus from eye margin, separated from median ocellus by approx. 2.0× its own diameter. Eyes with microsetae, which are approximately as long as width of facet. Frons microtrichose, without setae, flattened. Antennal length 1.1–1.3, 1.2 [1.3] mm (n=6). Face clearly longer than wide, setose; clypeus and labrum microtrichose, without setae. Palpus with four palpomeres; palpomere 1 oblong, without setae; other palpomeres with brown setae; palpomere 2 bearing small pocket of sensilla; palpomere 1 length longer than or subequal in length to palpomere 2; palpomere 3 approx. same length as combined length of palpomeres 1 and 2; palpomere 4 subequal or slightly longer in length to combined length of palpomeres 2 and 3.

*Thorax*. Dorsum with evenly-distributed, short, appressed setae, bearing longer setae only along lateral and posterior margins. Antepronotum, proepisternum, and laterotergite bearing setae; remaining lateral thoracic sclerites bare. Costal wing vein extends beyond R_5_, approx. three-fifths distance between R_5_ and M_1_; R_1_ approximately the same length as r-m or slightly longer; cubital fork below or proximad of r-m base (as in *Megophthalmidia occidentalis*, Fig. 52); R_1_, M_1_, M_2_, CuA_1_, and CuA_2_ with setae on upper surface (lacking setae on M_1_ + M_2_). Wing veins A1 and CuP absent.

*Male genitalia* (Figs 63–71). Posterior margin of epandrium broadly emarginate at center (Fig. 65). Posterior processes of epandrium relatively short, approx. 2× longer than wide, separated at base by approx. 1× width of process, turned inward (i.e., broad in lateral view (Fig. 63)); bare (Fig. 64). Gonocoxites as in Figs 66–68. Aedeagal fork bifurcated into tines; shorter tine slightly thickened (nearly same width as paired tine at base), both tines pointed outward apically (Fig. 70).

Female. Body length: 2.6–3.3, 2.8 mm (n=4). Antennal length: 0.8–1.0, 0.9 mm (n=4). Wing length: 2.5–3.1, 2.8 mm (n=4).

*Coloration* (Fig. 62). Same as male; cerci light brown to brown.

*Head and thorax*. Same as male, except antenna length shorter.

#### Etymology.

The species epithet “perignea” is an adjective, derived for the Latin word for “near fiery” in reference to the distribution of this species, relative to that of its sister taxon, *Megophthalmidia ignea* sp. n.

### Megophthalmidia radiata sp. n.

http://zoobank.org/2B07F97E-C352-4973-ADC1-F37178E9C494

http://species-id.net/wiki/Megophthalmidia_radiata

**Figure 72.**
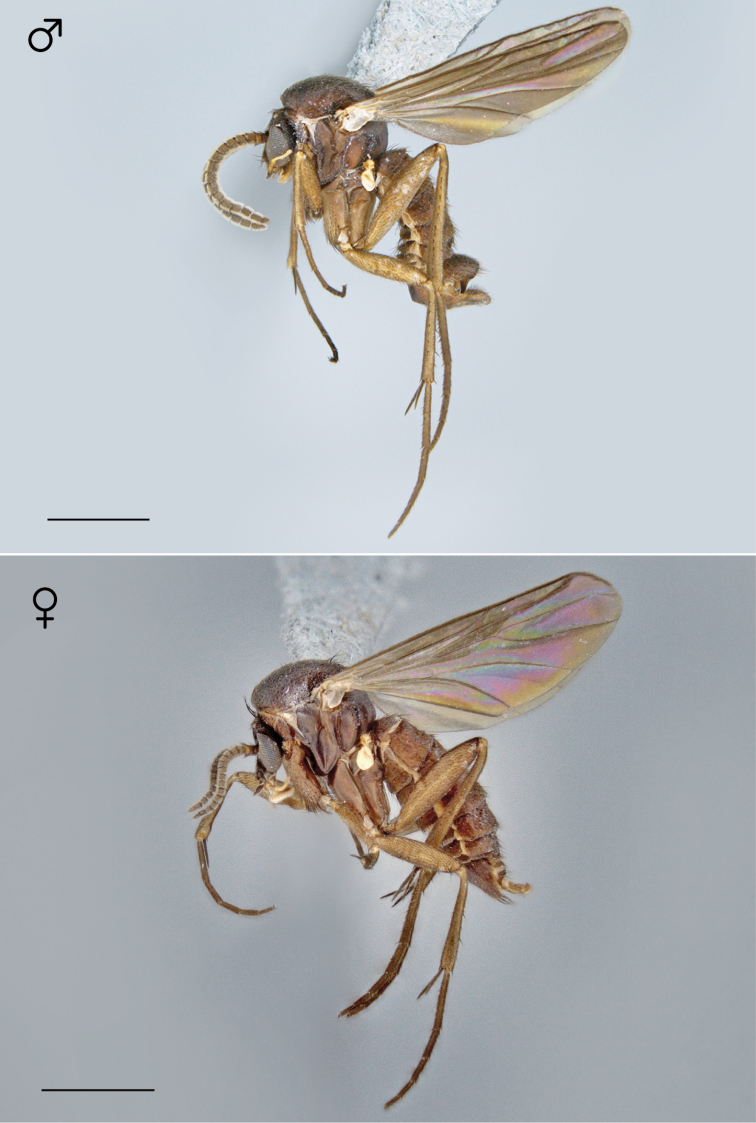
*Megophthalmidia radiata* sp. n., habitus [holotype male above, # 13M301; female below, # 13M344]. Scale bar = 1 mm.

Figs 72 [Fig F31] [Fig F32] –81

#### Type material.

Holotype: ♂, “USA: CA: San Luis Obispo Co., UC Rancho Marino Res., Malaise, 35.5391°N,-121.0790°W, 9–25.iv.2009 M.S. Caterino, CSCA12L333” / “HOLOTYPE 13M301, *Megophthalmidia radiata* ♂, Kerr, 2014” [red label]. Deposited in CSCA, mounted on gray point, missing mid and hind right legs, otherwise in excellent condition; specimen not dissected (Fig. 72).

Paratypes (all bearing blue paratype label): 3 ♂♂, ♀, same locality as holotype [SBMNH # 13M345 (♂); CSCA, specimen numbers 13M318 (dissected ♂, Figs 73–81), 13M343 (♂), 12M344 (♀, Fig. 72)]; ♂, “USA: CA: San Luis Obispo Co., UC Rancho Marino Res., Malaise, 35.5392°N,-121.0813°W, 15.iv–7.v.2009 M.S. Caterino CSCA13L250” [CSCA; # 13M787].

#### Diagnosis.

**Figures 73–75. F31:**
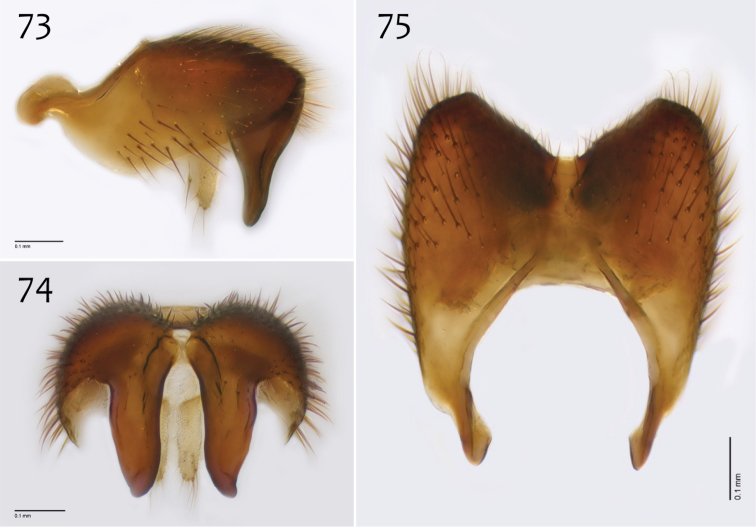
*Megophthalmidia radiata* sp. n., male epandrium [paratype, # 13M318] **73** Lateral view **74** Posterior view **75** Dorsal view. Scale bar = 0.1 mm.

*Megophthalmidia radiata* sp. n. may be confused with several Nearctic congeners that also have a brown thorax. Among these, it is most similar to *Megophthalmidia ignea*, *Megophthalmidia perignea*, and *Megophthalmidia misericordia* on account of having broad posterior epandrial processes. *Megophthalmidia radiata* has thicker posterior epandrial processes at their base than any of its congeners, including *Megophthalmidia ignea* and *Megophthalmidia misericordia* however, a character which is especially noticeable in lateral view (Fig. 73). The posterior epandrial processes are also very narrowly separated at their base (Fig. 74). The form of the adeagal complex is also diagnostic for this species (Figs 79–81).

#### Description.

Male. Body length: 2.6–2.9, 2.8 [2.9] mm (n=4). Wing length: 2.8–3.1, 3.0 [2.9] mm (n=4).

*Coloration* (Fig. 72). Male. Head dark brown; antennal scape, pedicel and flagellomeres brown to dark brown; face dark brown, clypeus and labrum brown to dark brown; palps and labellum cream-colored, pale yellow, to light brown (palpomeres 1–3 usually slightly darker than others, palpomere 2 with light patch where sensilla present). Thorax brown to dark brown throughout; scutum setae brown. Coxae nearly the same or lighter in color as thorax, light brown to brown, fore coxa same color as mid- and hind coxa; femora, tibia, and tarsi light brown to brown; hind tibial comb yellowish, preceded by 0–3 (usually 3) dark brown setae. Wing hyaline without markings, wing veins brown; haltere stem and knob cream-colored. Abdominal segments concolorous brown to dark brown. Terminalia brown.

*Head*. Ocelli slightly raised, median ocellus in line with anterior margin of lateral ocelli, median ocellus approx. 0.4–0.8× size of lateral ocelli; lateral ocellus located 1.5–2× diameter of ocellus from eye margin, separated from median ocellus by approx. same distance. Eyes with microsetae, which are approximately as long as width of facet. Frons microtrichose, without setae, flattened. Antennal length 1.5–1.6, 1.5 [1.5] mm (n=3). Face clearly longer than wide, setose; clypeus and labrum microtrichose, without setae. Palpus with four palpomeres; palpomere 1 oblong-triangular, without setae; other palpomeres with golden brown setae; palpomere 2 bearing small pocket of sensilla; palpomere 1 length longer than or subequal in length to palpomere 2; palpomere 3 length subequal to or slightly shorter than combined length of palpomeres 1 and 2; palpomere 4 length 0.7–1.2× combined lengths of palpomeres 1–3 In female, palpomere 4 appx. length of palpomeres 2–3.

*Thorax*. Dorsum with evenly-distributed, short, appressed setae, bearing longer setae only along lateral and posterior margins. Antepronotum, proepisternum, and laterotergite bearing setae; remaining lateral thoracic sclerites bare. Costal wing vein extends beyond R_5_, approx. two-thirds distance between R_5_ and M_1_; R_1_ approximately the same length as r-m or slightly longer; cubital fork below, slightly proximad or slightly distad of r-m base; R_1_, M_1_, M_2_, CuA_1_, and CuA_2_ with setae on upper surface (lacking setae on M_1_ + M_2_). Wing veins A1 and CuP absent.

**Figures 76–78. F32:**
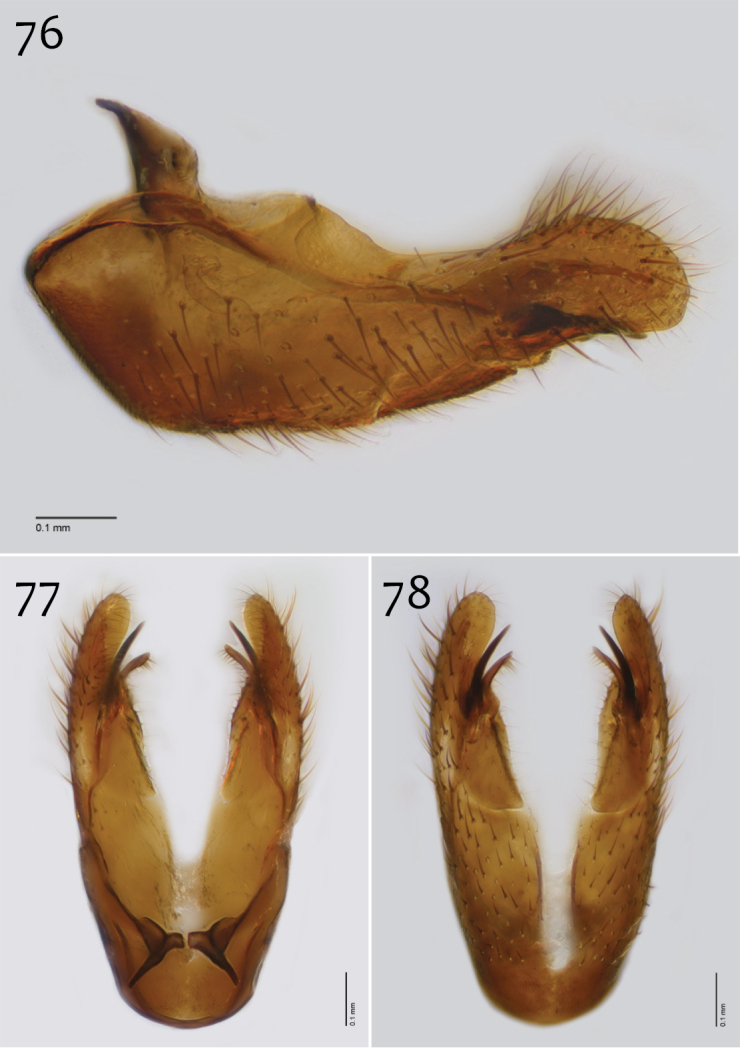
*Megophthalmidia radiata* sp. n., male hypandrium [paratype, # 13M318] **76** Lateral view **77** Dorsal view **78** Ventral view. Scale bar = 0.1 mm.

**Figures 79–81.**
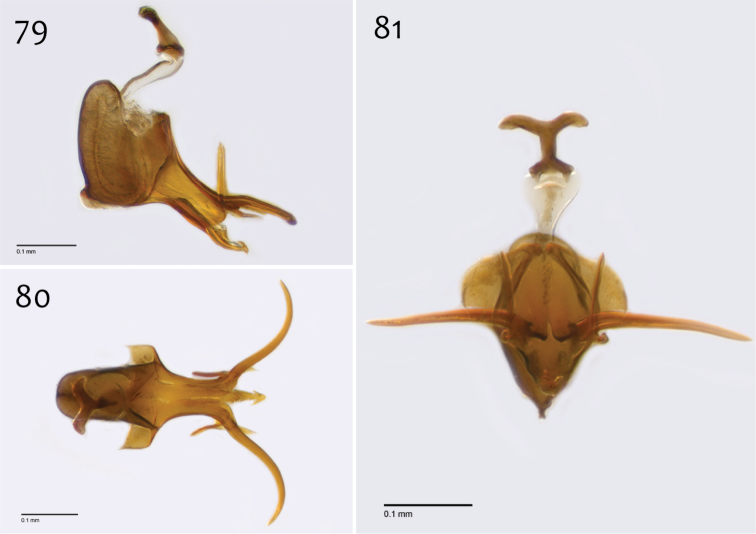
*Megophthalmidia radiata* sp. n., male aedeagus [paratype, # 13M318] **79** Lateral view **80** Dorsal view **81** Posterior view. Scale bar = 0.1 mm.

*Male genitalia* (Figs 73–81). Epandrium dorsal surface with medial depression, where setae are lacking; posterior margin broadly emarginate at center (Figs 74, 75). Posterior processes of epandrium broad, approx. 2.5× longer than narrowest width, narrowly separated at base, length of setae at base of epandrial processes approximately 0.5× width of process, bare along most of length (Figs 73, 74). Gonocoxites as in Figs 76–78. Aedeagal fork bifurcated into two tines one clearly longer (approx. 3×) and wider (approx. 2×) than the other; smaller tine pointed upward, longer tine s-curved, slightly recurved backward at apex (Figs 79–81).

Female. Body length: 3.2 mm (n=1). Wing length: 3.0 mm (n=1). Antennal length 1.1 mm (n=1).

*Coloration* (Fig. 72). Same as male, except abdominal segments 8–10 brown; cerci light brown to brown.

*Head and thorax*. Same as male, except antenna length shorter.

#### Etymology.

The species epithet “radiata” is a noun in apposition, due to the proximity of this species to *Pinus radiata* (Monterey Pine). The only known locality for this *Megophthalmidia* species, Kenneth S. Norris Rancho Marino Reserve (University of California Natural Reserve System), is one of only three areas where natural *Pinus radiata* forests still exist.

### Megophthalmidia saskia sp. n.

http://zoobank.org/D44EE8C8-5DBE-4437-BB1C-367790670A77

http://species-id.net/wiki/Megophthalmidia_saskia

**Figure 82.**
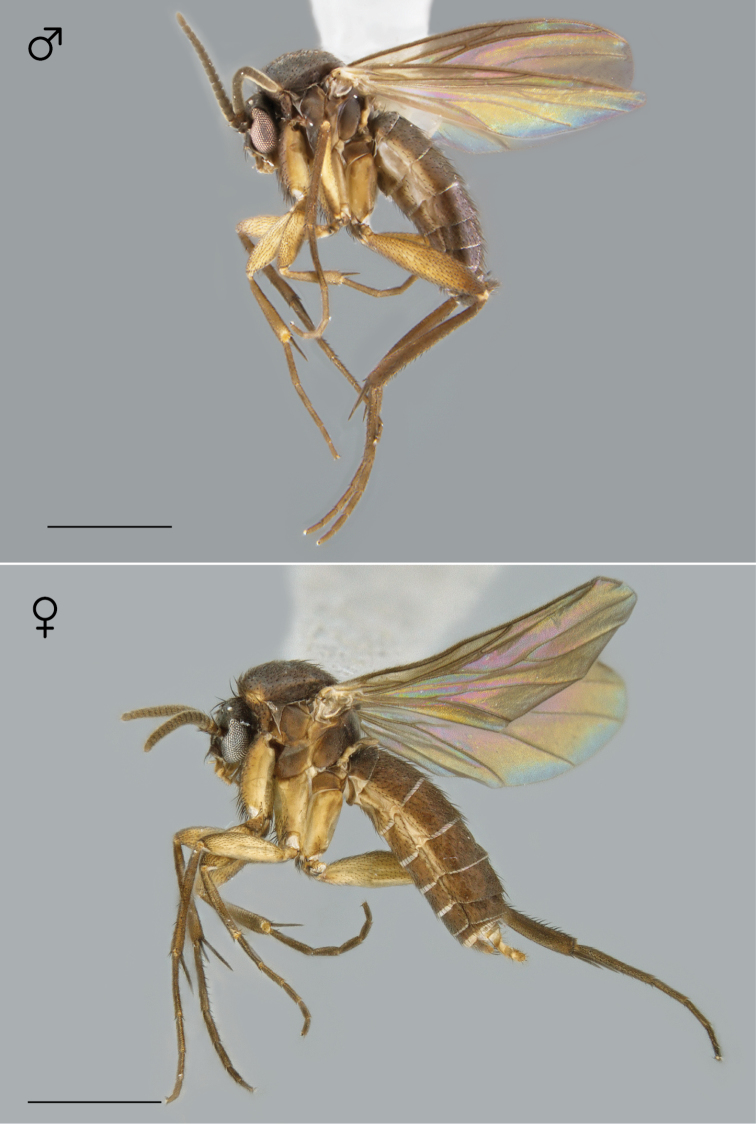
*Megophthalmidia saskia* sp. n., habitus [holotype male above, # 12J607; female below, # 13N400]. Scale bar = 1 mm.

Figs 82 [Fig F35] [Fig F36] [Fig F37] –92

#### Type material.

Holotype: ♂, “USA: CA: Marin: Pt. Reyes NS, LimantourRd, 2.6mi S BearVallRd, 2mMT, 38.0526°N,-122.8263°W, 13.iii–1.v.2012 P. H. Kerr, C. J. Borkent, CSCA12L022” / “HOLOTYPE 12J607, *Megophthalmidia saskia* ♂, Kerr, 2014” [red label]. Deposited in CSCA, complete specimen in excellent condition, mounted on gray point (Fig. 82). See Fig. 107 for image of type locality.

**Figure 83. F35:**
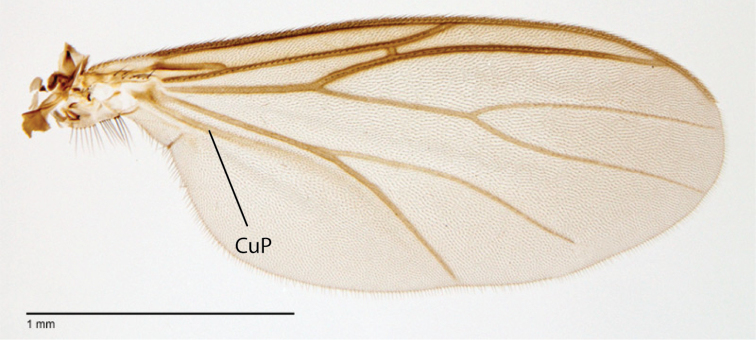
*Megophthalmidia saskia* sp. n., wing [paratype male, # 13M285]. Scale bar = 1 mm.

**Figures 84–86. F36:**
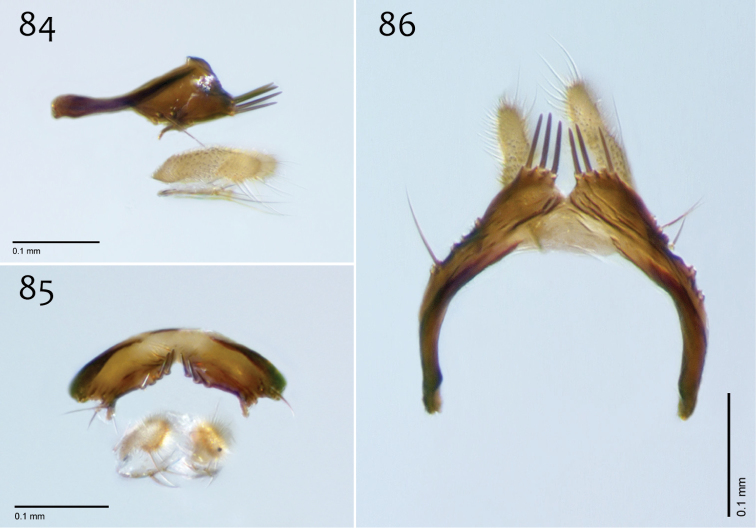
*Megophthalmidia saskia* sp. n., male epandrium [paratype, # 12J951] **84** Lateral view **85** Posterior view **86** Dorsal view. Scale bar = 0.1 mm.

**Figures 87–89. F37:**
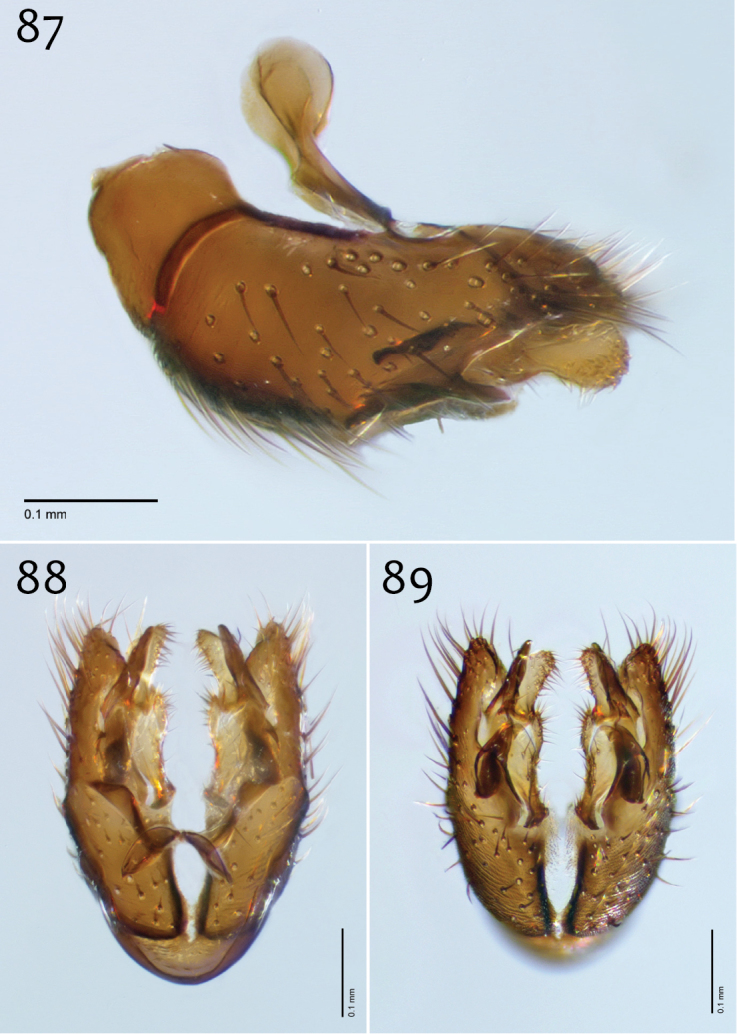
*Megophthalmidia saskia* sp. n., male hypandrium [paratype, # 12J951] **87** Lateral view **88** Dorsal view **89** Ventral view. Scale bar = 0.1 mm.

**Figures 90–92.**
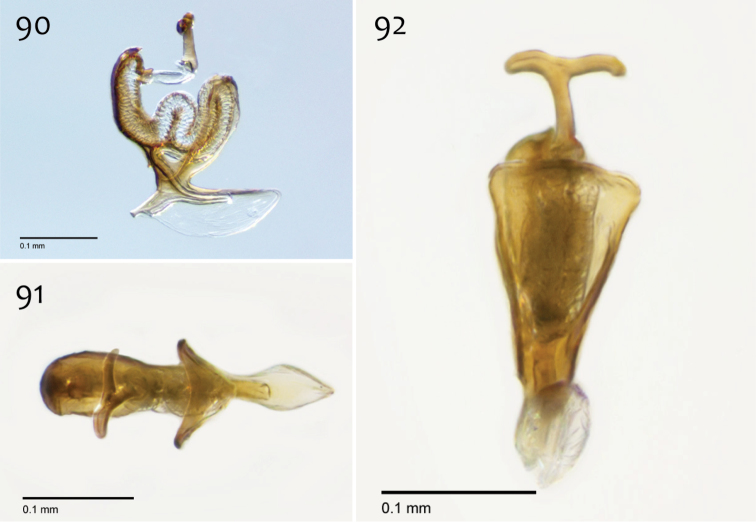
*Megophthalmidia saskia* sp. n., male aedeagus [paratype, # 12J951] **90** Lateral view **91** Dorsal view **92** Posterior view. Scale bar = 0.1 mm.

Paratypes: ♀, “USA: CA: Humboldt Co., Humboldt Bay NWR, Lanphere Dunes, MT#3 (6m), ~6masl, 40°53.421'N, 124°08.601'W 10.iv–18.viii.2008 P.H. Kerr, P.Haggard CSCA09L107” [CSCA; # 09E102]; 2 ♀♀, “USA: CA: Humboldt Co., Prairie Creek SP, Cal-Barrel Rd. trailhd, 41.3828°N, 123.9979°W, 300masl, 2.vi–25.vii.2009 P. Kerr & O. Lonsdale, 2mMT, CSCA09L519” [CSCA; numbers 09D513, 13N400 (Fig. 82)]; 2 ♂♂, 3 ♀ ♀ “USA: CA: Marin: Pt. Reyes NS, MtVisionRd, 1.8mi E SFDrakeBlvd 6mMT, 38.1013°N,-122.8878°W 280masl, 13.iii–1.v.2012, P. H. Kerr & C. J. Borkent, CSCA12L023” [CSCA; specimen numbers 12J951 (♂, Figs 84–92), 12J606 (♂), 12J450 (♀), 13N398 (♀),13N399 (♀)]; ♂, “USA: CA: Sonoma Co., Annadel SP, 0.9mi from park lot, Richardson trail, 38°26.11'N, 122°36.67'W 220masl, 6m MT#3, 17.v-7.vi.2007 P. Kerr & S. Blank, 07LOT196” [CSCA; # 07y537]; 2 ♂♂, “USA: CA: Marin: Pt. Reyes NS, MtVisionRd, 1.8mi E SFDrakeBlvd 6mMT, 38.1013°N,-122.8878°W 280masl, 13.iii–1.v.2012, P. H. Kerr & C. J. Borkent, CSCA12L023” [CSCA; # 12J578, # 13M285 (Fig. 83); both in alcohol]; ♂, “USA: CA: Sonoma Co., Annadel SP, 0.9mi from park lot, Richardson trail 38°26.11'N, 122°36.67'W 220masl, 6m MT, 3–26.iv.2007 P. Kerr & S. Blank 07LOT029” [Locality Fig. 103; CSCA; # 12K736, in alcohol].

#### Diagnosis.

*Megophthalmidia saskia* is easily separated from its putative congeners by its having a setose frons, wing vein CuP (Fig. 83), dark haltere, and distinctive genitalia (Figs 84–89).

#### Description.

Male. Body length: 2.5–2.7, 2.6 [2.6] mm (n=3). Wing length: 2.5–2.7, 2.6 [2.6] mm (n=3).

*Coloration* (Fig. 82). Head, antennae, face, clypeus and labrum dark brown to black; palps and labellum light brown to brown. Thorax dark brown to black throughout; scutum setae black. Coxae light brown to brown, anterior surface of fore coxae darker; femora becoming gradually darker dorsoapically, tibiae brown to dark brown (hind tibia darkest), tarsi brown; hind tibial comb dark brown, preceded by one longer dark brown seta. Wing hyaline without markings, wing veins dark brown; haltere stem and knob dark brown. Abdominal segments concolorous dark brown to black, except sternites 1–3 usually paler brown. Terminalia dark brown to black.

*Head*. Ocelli slightly raised, not arranged in a line (median ocellus clearly anterior of lateral ocelli); median ocellus very small, approx. 0.2× size of lateral ocelli; lateral ocellus located approx. 3× diameter of ocellus from eye margin, separated from median ocellus by approx. twice its own diameter. Frons microtrichose and setose, flattened. Eyes with microsetae, which are approximately as long as width of facet. Antennal length 1.0–1.1, 1.0 [1.1] mm (n=3) (approx. 1× length of head and thorax). Face nearly as wide as long, microtrichose, bearing black setae medially and along ventral margin; clypeus microtrichose, without setae; labrum microtrichose, without setae. Palpus with four palpomeres; palpomere 1 barrel-shaped, without setae; other palpomeres with golden brown to dark brown setae; palpomere 2 bearing small pocket of sensilla; palpomere 1 subequal in length to palpomere 2; palpomere 3 length slightly shorter than combined length of palpomeres 1 and 2; palpomere 4 length 0.75–1× combined lengths of palpomeres 1–3.

*Thorax*. Dorsum with evenly-distributed, short, appressed setae, bearing longer setae only along lateral and posterior margins. Antepronotum, proepisternum, and laterotergite bearing setae; remaining lateral thoracic sclerites bare. Wing venation as in Fig. 83; costal vein extends beyond R_5_, approx. two-thirds distance between R_5_ and M_1_; R_1_, M_1_, M_2_, CuA_1_, and CuA_2_ with setae on upper surface (lacking setae on M_1_ + M_2_). CuA_1_ usually reaching wing margin (Fig. 83 shows irregularity in this respect). Wing vein A1 absent, CuP present as prominent fold with at least some apparent brown coloration (sclerotization).

Dorsum with evenly-distributed, short, appressed setae, bearing longer setae only along lateral and posterior margins. Antepronotum, proepisternum, and laterotergite bearing setae; remaining lateral thoracic sclerites bare. Costal wing vein extends beyond R_5_, approx. two-thirds distance between R_5_ and M_1_; cubital fork below, at same level or distad of r-m base; R_1_, M_1_, M_2_, CuA_1_, and CuA_2_ with setae on upper surface (lacking setae on M_1_ + M_2_).

*Male genitalia* (Figs 84–92). Epandrium narrow laterally, without setae medially, narrowly emarginate at center, with posterior margin bearing 6–8 posteriorly-directed strong setae, lacking posterior processes (Figs 84–86). Gonocoxites with anteroapical expansion, centrally-attached dorsal apodeme and bearing short ventral process (Figs 87–89). Aedeagus reduced, without aedeagal fork (Figs 90–92).

Female. Body length: 2.8–3.5, 3.2 mm (n=6). Antennal length: 0.7–1.1, 0.9 mm (n=5). Wing length: 2.6–3.5, 3.1 mm (n=6).

*Coloration* (Fig. 82). Same as male; cerci light brown to brown.

#### Etymology.

The species name “saskia,” a noun in apposition, is given in honor of my daughter, Saskia Ana Kerr, born April 20, 2013. Just as Saskia is to our family, *Megophthalmidia saskia* is a special addition to this group.

#### Discussion.

The male terminalia of *Megophthalmidia saskia* is unlike any other *Megophthalmidia* species in North America. Furthermore, *Megophthalmidia saskia* shares a number of characters with *Mohelia* and *Aphrastomyia* that until now, have not been observed in *Megophthalmidia*. These characters include wing vein CuP present and frons bearing setae. As such, *Megophthalmidia saskia* may be an important entity for understanding character evolution across the group containing *Megophthalmidia*, *Mohelia*, and *Aphrastomyia*. *Megophthalmidia* found elsewhere (e.g., *Megophthalmidia crassicornis* (Curtis) of Europe and *Megophthalmidia divergens* Edwards of the neotropics) show a degree of variation that implicitly defines the genus as a dumping ground of putatively related species that are not *Aphrastomyia*. *Mohelia* is also related, but its status is poorly understood and representative specimens were not available for this study. While *Megophthalmidia saskia* clearly represents a distinct evolutionary divergence, it is premature to construct additional genus-level concepts until the entire group is studied in detail, within a broader phylogenetic and biogeographic context. Such work is in development and ongoing.

### Ectrepesthoneura marceda

(Sherman, 1921) comb. n.

http://species-id.net/wiki/Ectrepesthoneura_marceda

**Figure 93.**
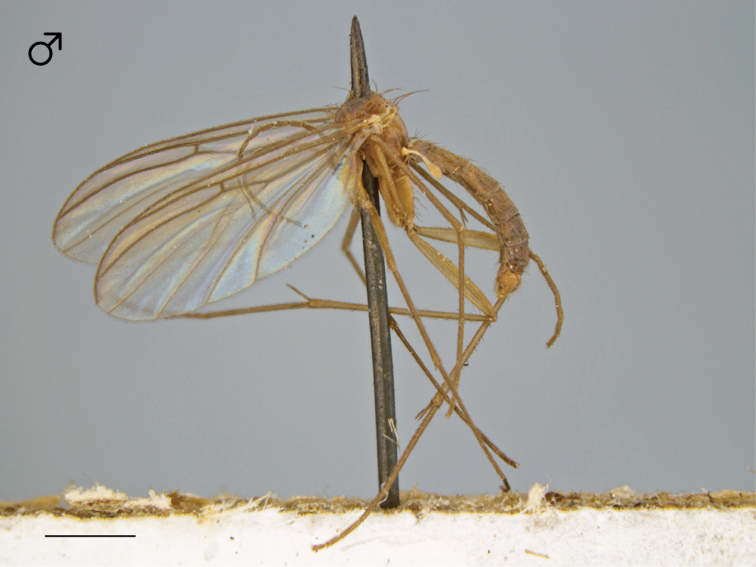
*Ectrepesthoneura marceda* (Sherman), habitus [male specimen # **598**; 12K405]. Scale bar = 1 mm.

Figs 93 [Fig F40] [Fig F41] –102

Tetragoneura marceda Sherman, 1921: 20

#### Examined material.

Although the original description states that 35 specimens were examined, the syntype series currently consists of 22 specimens labeled as “Tetragoneura marceda Sherman, 1921” within the CNC collection. One specimen is a female *Mycetophila* sp. (specimen # 765). As an additional note, it appears that C.B. Garrett added type labels and notes on Sherman’s labels (e.g., “R.2.3. absent, ♂”). Specimens # 600 and # 324 have vein R4 present on the right wing. E.I. Coher dissected one specimen (specimen # 473) and labeled it as the holotype. As first reviser and in the interests of stable taxonomy, I designate this specimen as the lectotype.

Lectotype: ♂, “Savary Id., 10.4.17 [on mount] / 473 [on back side of mount]/ R.2.3 ABSENT, ♂, Tetragoneura marceda R.S. Sherman, ♂ / TYPE, See letter [handwritten], C.B.D. Garrett / HOLOTYPE, Megophtalmidia [sic] marceda, (Sherman) 1921 [“(Sherman) 1921” on underside; handwritten label created by E.I. Coher] / Megophtalmidia [sic] marceda (Sherman) 1921, ’98 EIC [handwritten label created by E.I. Coher] / LECTOTYPE, Ectrepesthoneura marceda (Sherman, 1921), P.H. Kerr, xii.2013” [CNC]; double-mounted specimen, terminalia dissected, otherwise complete; genitalia vial pinned beneath specimen.

**Figures 94–95. F40:**
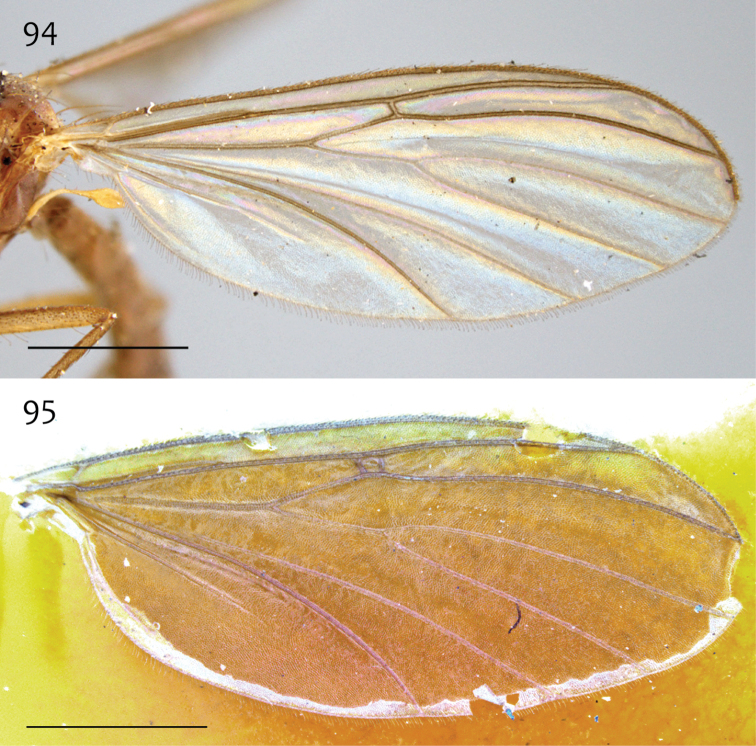
*Ectrepesthoneura marceda* (Sherman), wing **94** Specimen # 616; 13M406 **95** Specimen # 329; 13M409. Scale bar = 1 mm.

**Figures 96–98. F41:**
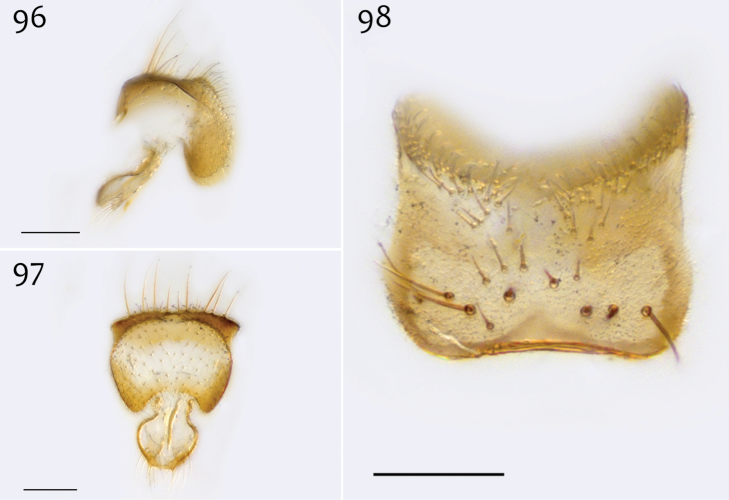
*Ectrepesthoneura marceda* (Sherman), male epandrium [paralectotype specimen #324; 13M411] **96** Lateral view **97** Posterior view **98** Dorsal view. Scale bar = 0.1 mm.

**Figures 99–102.**
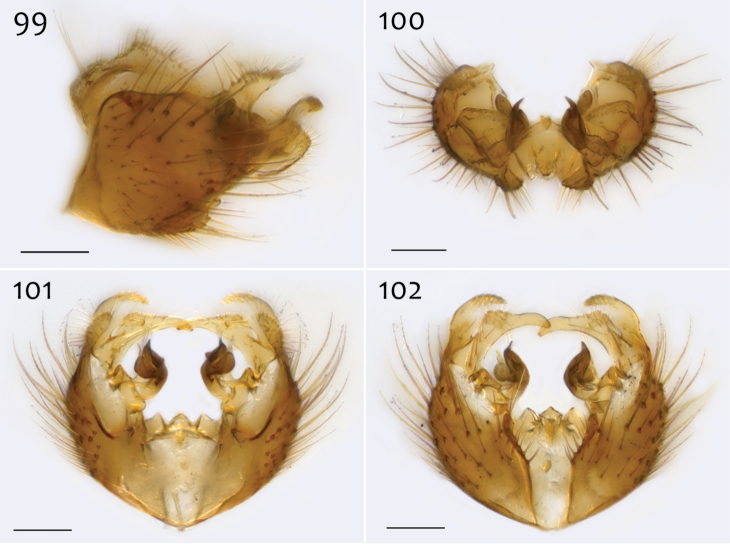
*Ectrepesthoneura marceda* (Sherman), male hypandrium [paralectotype specimen #324; 13M411] **99** Lateral view, with epandrium **100** Posterior view **101** Dorsal view **102** Ventral view. Scale bar = 0.1 mm.

Paralectotypes [CNC; all specimens include additional Sherman and Garrett labels similar to that of the lectotype except specimen # 573 which lacks Garrett label and specimen # 463, which does not have any additional labels]: 5 ♂♂, ♀, “Savary Id., 10.4.17 [handwritten on mount]” specimen numbers indicated on the back of each mount: 486 (♂), 520 (♂), 598 (♂, #12K405, Fig. 93), 600 (♂), 616 (♂, Fig. 94), 463 (♀); 3 ♂♂, “Savary Id., 7.4.17 [handwritten on mount]” specimen numbers indicated on the back of each mount: 324 [dissected 13M411, Figs 96–102], 329 [specimen destroyed, wing glued to card; Fig. 95], 457 [below crossed-out “332”; specimen glued directly to side of card]; 2 ♂♂, “Savary Id. B.C., 7.4.17 [handwritten on mount]” specimen numbers indicated on the back of each mount: 436 [below crossed-out “311”], 450 [below crossed-out “325”]; ♂, “Savary Id., 9.4.17 [handwritten on mount] / 405 [back of mount]”; ♀, “Savary Id., 10.4.17 [handwritten on mount] / 573 [back of mount]” [dissected]; 2 ♂♂, “Savary Id., 11.4.17 [handwritten on mount]” specimen numbers indicated on the back of each mount: 664, 669; 4 ♂♂, “Savary Id., 15.4.17 [handwritten on mount]” specimen numbers indicated on the back of each mount: 733, 742, 758, 759; ♀, “Stanley Park, 20.v.17 B.C. [handwritten on mount] / 1393 [back of mount]”.

**Figures 103–108.**
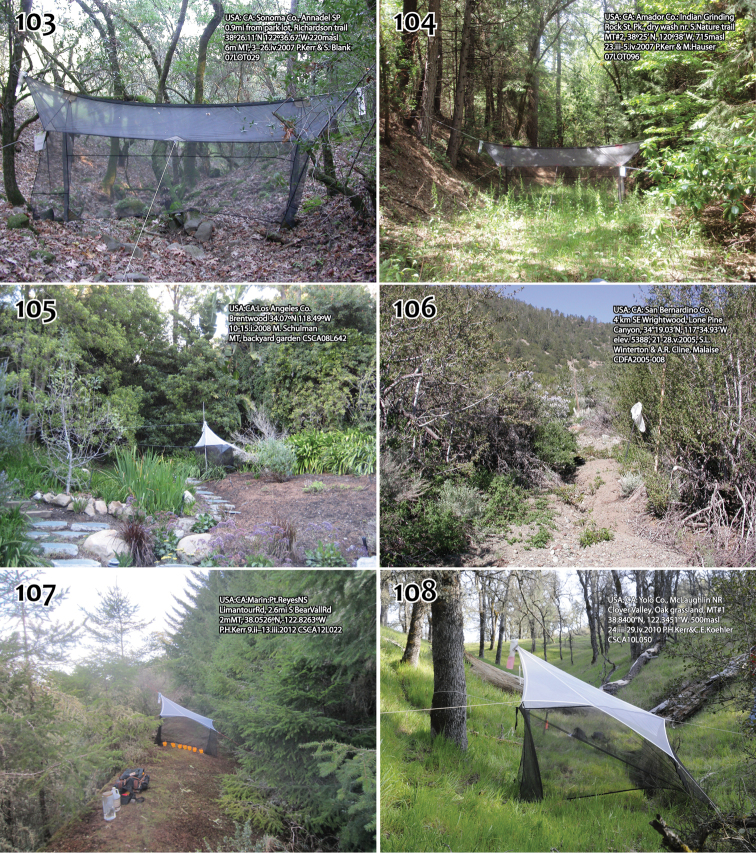
*Megophthalmidia* localities, with locality lot numbers in brackets **103** Annadel State Park [07LOT029] **104** Indian Grinding Rock State Historical Park [07LOT096] **105** Backyard garden, Brentwood, CA [CSCA08L642] (Photo courtesy of B.V. Brown) **106** Lone Pine Canyon, Wrightwood, CA [CDFA2005-008] (Photo courtesy of S.L. Winterton) **107** Point Reyes National Seashore [CSCA12L022] **108** University of California McLaughlin Natural Reserve [CSCA10L050].

## Conclusions

Although most North American species of *Megophthalmidia* may be distinguished by external features of the male genitalia (viz. the posterior epandrial processes), the aedeagal complex is especially informative for species recognition and critical for distinguishing closely related species such as *Megophthalmidia occidentalis*/*Megophthalmidia mckibbeni* and *Megophthalmidia ignea*/*Megophthalmidia perignea*. It should be encouraged that future taxonomic contributions clearly illustrate the aedeagal complex in this and related genera.

More collecting efforts are needed to discover additional areas inhabited by *Megophthalmidia* species in California and the rest of Western North America. For now, several new species are known only from areas restricted within the California State Park and the UC California Natural Reserve systems. As is evident in this study and others (Myers et al. 2000; Connor et al. 2003; Caterino et al. 2008; Chandler and Caterino 2011; Fiedler et al. 2013), these parks and reserves are critically important for the continued discovery and appreciation of our natural heritage and merit greater attention and funding support.

## Supplementary Material

XML Treatment for Megophthalmidia browni

XML Treatment for Megophthalmidia ignea

XML Treatment for Megophthalmidia lenimenta

XML Treatment for Megophthalmidia mckibbeni

XML Treatment for Megophthalmidia misericordia

XML Treatment for Megophthalmidia occidentalis

XML Treatment for Megophthalmidia perignea

XML Treatment for Megophthalmidia radiata

XML Treatment for Megophthalmidia saskia

XML Treatment for Ectrepesthoneura marceda
